# Novel insights into β3-adrenoceptor function in a model of retinopathy of prematurity: the contribution of astrocytes to retinal protection

**DOI:** 10.3389/fnins.2026.1891523

**Published:** 2026-08-19

**Authors:** Alberto Melecchi, Alessio Canovai, Lorenza Di Marsico, Rosario Amato, Massimo Dal Monte, Luca Filippi, Paola Bagnoli, Maurizio Cammalleri

**Affiliations:** 1 Department of Biology, University of Pisa, Pisa, Italy; 2 Center for Instrument Sharing of the University of Pisa (CISUP), University of Pisa, Pisa, Italy; 3 Interdepartmental Center Pisa Neuroscience “PiNeuro, ” University of Pisa, Pisa, Italy; 4 Neonatal Intensive Care Unit, Meyer Children's Hospital IRCCS, Florence, Italy

**Keywords:** astrocyte recovery, blood retinal barrier, electroretinography, neovascularization, neurotrophic factors, retinal ganglion cells, vaso-obliteration

## Abstract

The β-adrenoceptor (β-AR) system has been implicated in the pathological angiogenesis of the retina. Of the three β-ARs, the role of β3-AR remains to be elucidated. In a mouse model of oxygen-induced retinopathy (OIR), we investigated the role of β3-ARs using the 129S inbred mouse strain, which is highly responsive to hypoxia because of its marked imbalance between pro- and anti-angiogenic factors. We found that β3-AR activation with BRL37344 and CL316243, both potent, first-generation β3-AR agonists promotes the revascularization of the central retina in association with the restored astrocyte template. Recovered vascularization of the central retina prevents the onset of hypoxia thus impeding the activation of the pro-angiogenic pathway and inhibiting neovessel overgrowth in the mid periphery, blood-retinal barrier leakage and retinal dysfunction. Vessel rescue and recovered astrocyte density were found to be associated with protective effects against the OIR-related loss of retinal ganglion cells (RGCs) as also demonstrated by their recovered functional activity. RGC rescue occurs in connection with restored availability of neurotrophic factors that are otherwise impaired under hypoxia. Results from microdissected astrocytes were indicative of the possibility that astrocytes might contribute to retinal rescue. β3-AR has emerged as a potential intermediary in hypoxia-dependent neovascularization, thereby providing a rationale for exploring the causality between astrocyte recovery and retinal rescue with the aim of underlining the therapeutic potential of β3-AR agonists in proliferative retinopathies.

## 1 Introduction

Retinal vessel proliferation in response to ischemic damage, a major cause of severe visual impairment, can be mimicked by the model of oxygen induced retinopathy (OIR), which besides recapitulating retinopathy of prematurity (ROP), can be extended to much

more widely diffused retinal pathologies characterized by neovessel overgrowth. The OIR model includes two phases that follow one another. The first hyperoxic phase is characterized by vessel loss in the central retina, which leads to low oxygen tension causing a second phase characterized by disorganized, neovascular regrowth in the midperiphery through the activation of a pro-angiogenic cascade leading to endothelial cell recruitment causing aberrant neovascularization (Scott and Fruttiger, 2010). During this phase, neovessel overgrowth is associated with blood-retinal barrier (BRB) breakdown, which contributes to dysfunctional retinal activity as determined by electroretinogram (ERG) recordings (Shan et al., 2023). The central avascular area is strongly associated with retinal ganglion cell (RGC) loss as a result of the non-perfused central region that creates an ischemic environment triggering excitotoxic damage to RGCs and/or oxidative stress (Kaur et al., 2008; Yang et al., 2009).

In addition to directly activating the transcription factor hypoxia-inducible factor 1 (HIF-1) leading to vascular endothelial growth factor (VEGF) upregulation, hypoxia overactivates the sympathetic nervous system leading to an increased release of norepinephrine (NE) by sympathetic nerve fibers innervating the choroidal vessels from which it reaches the retina by paracrine diffusion (Dal Monte et al., 2012; Ruan et al., 2020).

In the retina, NE activates beta adrenoceptors (β-ARs) of which β3-AR is increasingly emerging as an attractive target for drug discovery. β3-AR is characterized by a unique pharmacological profile, including the lack of phosphorylation sites that render it less inclined to be desensitized through internalization/degradation (Okeke et al., 2019; Michel et al., 2020) and selective coupling to cAMP- and NO/cGMP-associated pathways (Samanta et al., 2024). Its low sensitivity to catecholamine renders β3-AR well responsive to sympathetic overstimulation when the high affinity brothers are desensitized after prolonged NE release (Michel et al., 2020). β3-AR lower sensitivity to β1/β2-AR ligands differentiates it from the other β-AR subtypes. These pharmacological properties, together with growing evidence of its involvement in retinal angiogenesis, have raised great interest in preclinical studies aimed at developing future strategies for chronic retinal diseases (Böhm et al., 2023; Cammalleri et al., 2023; Pasha et al., 2024). In respect to its potential involvement in retinal angiogenesis, previous experimental studies in cultured endothelial cells have demonstrated that β3-AR activation can promote pro-angiogenic responses including enhanced cell migration, proliferation and tube formation (Steinle et al., 2003, 2005).

In the OIR model, β3-AR is upregulated in response to hypoxia in association with the accumulation of HIF-1α (Ristori et al., 2011) and the recent demonstration that β3-AR acts as a novel HIF-1 target gene, suggests its potential role as an intermediary between oxygen levels and retinal vessel proliferation (Amato et al., 2022). While β3-AR is emerging as a promising target for pharmacotherapy of several pathologies, its potential role in the retina remains to be determined. In fact, previous findings in the OIR model of the C57BL/6J (C57), a common inbred strain of laboratory mouse, failed to reveal any significant effect of β3-AR pharmacology on OIR-associated neovascularization (Martini et al., 2011).

The aim of this work was to provide a first evaluation of a possible regulatory role of β3-AR in hypoxia-driven retinal vessel proliferation using the OIR model in the 129S3/SvIM (129S) inbred strain, which is highly responsive to hypoxia because of its marked imbalance between pro- and anti-angiogenic factors (Chan et al., 2005). An additional advantage of the 129S strain is its drastic overexpression of β3-AR messenger in the hypoxic retina, which makes it well-suited to study the role of β3-AR in the angiogenic response to hypoxia (Chen et al., 2012).

In the OIR model, we evaluated the efficacy of β3-AR activation on hypoxia-associated retinal damages including neovessel proliferation, BRB dysfunction, astrocyte impairment and RGC loss. Whether β3-AR activation might influence OIR-driven retinal dysfunction was also investigated by electroretinographic recordings. Considering the importance of the astrocyte template in retinal angiogenesis (Paisleyn and Kay, 2021), the possibility that β3-AR agonism might prevent OIR-associated vascular impairment through recovered astrocytes was additionally investigated. Whether β3-AR activation might intervene in the interplay between RGCs and astrocytes was also studied by determining its potential efficacy on RGC resilience and neurotrophic factor availability. To further elucidate the contribution of astrocytes to the protective effect of β3-AR activation, in microdissected astrocytes from the retinas of OIR mice, we determined the potential role of β3-AR activation on hypoxia-associated astrocyte impairment.

Early work by Melecchi et al. (2024) suggested an important role of β3-AR agonism in preventing hypoxia-induced angiogenesis in the OIR model. While its findings have been retracted (Melecchi et al., 2025), their implication for emerging pharmacological treatments provided us with some opportunities for replicating some of these results and implementing them by investigating the mechanisms underlying the β3-AR role in the retina including the contribution of astrocytes to the β3-AR-mediated protection of the hypoxic retina.

## 2 Materials and methods

### 2.1 Animals

Mice of both sexes in the 129S strain and the C57 strain were purchased from Charles River Laboratories, Italia (Calco, Italy). In the FVB/N genetic background, β3-AR–/– mice were purchased from The Jackson Laboratory (Bar Harbor, ME, USA). Mice were mated in our breeding colonies. They were housed in a controlled environment (23 ± 1 °C, 50 ± 5% humidity) with a 12 h light/dark cycle (lights on at 8:00 am.) and had free access to food and water. All procedures were approved by the Italian guidelines for animal care (DL 26/14, permission number: 132/2019-PR). They adhered to the ARVO Statement for the Use of Animals in Ophthalmic and Vision Research, the Italian animal care guidelines (DL 26/14), and the EU Directive (2010/63/EU). Overall, 201 mice were used. Of them, 174 mice of the 129S strain included 126 mice that were used at postnatal day (PD) 17, 24 mice at PD12 and 24 mice at PD15. In the C57 strain, 24 mice were used at PD17. In the FVB/N genetic background, 3 mice were used at PD17.

### 2.2 Experimental model of OIR and pharmacological treatments

In the mouse model of OIR (Smith et al., 1994), litters of mouse pups and their nursing mothers were placed in an incubator and exposed to high oxygen levels (75 ± 2%) from PD7 to PD12, before returning to room air between PD12 and PD17 (normoxia sensed as relative hypoxia). Oxygen levels were monitored twice daily with an oxygen analyzer (Pro-Custom Elettronica, Milano, Italy). The number of pups per litter used for OIR experiments was kept small (5–7 pups), and the body weight was monitored to exclude mouse pups weighing < 5 g at PD17. BRL37344 and CL316243 (Sigma-Aldrich, St. Louis, MO, USA), two β3-AR agonists, and SR59230A (Sigma-Aldrich), a β3-AR antagonist were dissolved in dimethylsulfoxide (Sigma-Aldrich) diluted in sterile saline (at a maximal concentration of less than 0.1%) as a vehicle. BRL37344 (2 mg kg^−1^) and CL316243 (1 mg kg^−1^) were administered daily by subcutaneous injections from PD12 to PD16. The effect of BRL37344 and CL316243 was tested at PD17. In order to determine the selectivity of BRL37344 and CL316243 at β3-AR, SR59230A, at a concentration of 20 mg kg^−1^, was administered from PD12 to PD16, 6 h before the injection of either BRL37344 or CL316243 and its effect was tested at PD17. In additional experiments, the efficacy of BRL37344 on HIF-1-associated β3-AR expression was also determined over the hypoxic phase (from PD12 to PD17).

BRL37344 and CL316243 were used in previous works at a dosage ranging from 1 to 3 mg kg^−1^ for BRL37344 (Pott et al., 2003; Filippi et al., 2023) and from 0.5 to 1 mg kg^−1^ for CL316243 (Xiao et al., 2015; Tournissac et al., 2021) both including the dosage used in the present study. In particular, the dosage of BRL37344 used here was previously utilized in the OIR model (Dal Monte et al., 2015). In addition, SR59230A was used at a concentration of 20 mg kg^−1^ in line with previous results in the OIR model (Martini et al., 2011). The efficacy of subcutaneous administration of β-AR ligands was determined by previous findings demonstrating their effectiveness at the receptor level (Martini et al., 2011; Dal Monte et al., 2015). In the case of the OIR model, the subcutaneous route of administration is less invasive and can be easily repeated over the hypoxic phase even in the mouse pups.

In the present study, all experiments were performed at the same time of day to exclude possible circadian influences. The data were collected from both males and females and the results were combined as there was no apparent sex difference in line with previous studies (Higgins et al., 2001). No sex difference was found between males and females in terms of their response to β-AR ligands. In all Figures, BRL37344, CL316243 and SR59230A are referred to as BRL, CL and SR, respectively.

### 2.3 Microdissection of retinal astrocytes

In some experiments aimed at evaluating whether astrocytes are involved in the β3-AR-driven response to hypoxia, enzyme-assisted microdissection was used to mechanically isolate astrocytes from their location at the vitreoretinal surface as detailed by Cullen et al. (2023). Briefly, after euthanasia, the eyes from PD17 mice either normoxic or OIR, untreated or treated with BRL37344 were enucleated and the retina dissected. Retinal whole mounts were placed on a filter paper with RGCs facing up. A glass coverslip was flattened onto the vitreal surface and the collected pile of paper, retina and glass was reversed. After a collagenase solution (150 U/ml, Type II Collagenase, C2-BIOC; Sigma-Aldrich) was applied onto the filter paper, a weight of about 200 mg was placed above the pile which was then incubated for 20–25 min at 37 °C in a Petri dish to prevent drying. The coverslip was then gently removed by gradual lifting to prevent morphological alterations of the potentially isolated astrocytes, which were then processed for immunostaining or RNA isolation.

### 2.4 Isolectin B4 staining of retinal vessels

Dissected whole mount retinas were fixed by immersion in 4% w/v paraformaldehyde in 0.1 M phosphate-buffered saline (PBS) for 2 h at 4 °C. After fixation, samples were transferred into a 25% w/v sucrose solution in 0.1 M PBS and stored at 4 °C until used for isolectin B4 (IB4) staining or immunofluorescence analyses (see below). To visualize retinal vasculature, retinas were incubated with FITC-conjugated IB4 (1:200; Vector Laboratories, Burlingame, CA, USA) diluted in 2% v/v Triton X-100 in PBS for 48 h at 4 °C. After being rinsed in PBS, retinas were flat mounted on polarized glass slides with the ganglion cell layer (GCL) up. For each whole mount, image-editing software (ImageJ; National Institutes of Health, Bethesda, MD) was used to quantify retinal avascular area and tuft area as calculated in pixels and expressed as a percentage of the total retinal area. Retinal vasculature was also observed in whole mount retinas with an epifluorescence microscope (Ni-E; Nikon-Europe, Amsterdam, The Netherlands) equipped with a 10x Plan-Apochromat objective and a digital camera (DS-Fi1c camera, Nikon-Europe) or confocal microscopy (ECLIPSE 80i fluorescence microscope, Nikon-Europe). Confocal optical sections of the superior vascular plexus were obtained by scanning acquisition with a 10x objective and a detector resolution of 1024 × 1024 pixels. To test for vessel presence in microdissected astrocytes potential IB4 staining was observed in confocal microscopy.

### 2.5 Western blotting

Retinas were lysed in RIPA lysis buffer (Santa Cruz Biotechnology, Dallas, TX, USA) supplemented with proteinase and phosphatase inhibitor cocktails (Roche Applied Science, Indianapolis, IN, USA). The protein concentration was evaluated by the Micro BCA Protein Assay (Thermo Fisher Scientific, Waltham, MA, USA). Subsequently, 30 μg of protein from each sample was separated using SDS-PAGE gels (4–20%; Bio-Rad Laboratories, Hercules, CA, USA) and the gels were then transblotted onto nitrocellulose membranes (Bio-Rad Laboratories). After being blocked with 5% w/v skim-milk in 1x Tris-Buffered Saline containing 0.05% Tween-20 for 1 h at room temperature, the membranes were incubated overnight at 4 °C with the primary antibodies listed in Table 1. Thereafter, membranes were incubated for 2 h at room temperature with appropriate horseradish peroxidase-conjugated anti-rabbit (170-6, 515, Bio-Rad Laboratories) or anti-mouse (A9044, Sigma-Aldrich) secondary antibodies (dilution:1:5,000). Blots were developed using the Clarity Western enhanced chemiluminescence substrate (Bio-Rad Laboratories) and blot images were acquired using ChemiDoc XRS+ (Bio-Rad Laboratories). The optical density (OD) relative to the target bands was measured using Image Lab 3.0 software (Bio-Rad Laboratories) and normalized to the corresponding OD of β-actin as a loading control. For each experimental condition, 6 independent samples, each from 2 retinas of 2 different mice were used.

**Table 1:** Primary antibodies used in the Western blot analyses.

Antibodies	Dilution	Source	Cat. no
Mouse monoclonal anti-β3-AR	1:100	Santa cruz biotech	sc-515763
Rabbit monoclonal anti-VEGFA	1:500	Abcam	ab214424
Rabbit monoclonal anti-HIF-1α	1:1,000	Abcam	ab179483
Rabbit polyclonal anti-PEDF	1:1,000	Abcam	ab180711
Rabbit polyclonal anti-ZO-1	1:500	Invitrogen	40-2200
Mouse monoclonal anti-claudin-5	1:500	Invitrogen	35-2500
Rabbit monoclonal anti-GFAP	1:5,000	Abcam	ab207165
Rabbit monoclonal anti-PAX2	1:1,000	Abcam	ab79389
Rabbit monoclonal anti-CNTF	1:1,000	Abcam	ab270992
Rabbit monoclonal anti-BDNF	1:1,000	Abcam	ab108319
Mouse monoclonal anti-β-actin	1:2,500	Sigma-aldrich	A2228

### 2.6 Immunofluorescence

Retinal whole-mounts and microdissected astrocytes were rinsed in 0.1 M PBS and incubated for 72 h at 4 °C with the primary antibodies listed in Table 2. Primary antibodies were diluted in 0.1 M PBS containing 2% v/v Triton X-100.

**Table 2:** Primary antibodies used in immunofluorescence.

Antibodies	Dilution	Source	Cat. No
Rabbit monoclonal anti-GFAP	1:400	Abcam	ab207165
Rabbit monoclonal anti-PAX2	1:1,000	Abcam	ab79389
Rabbit polyclonal anti-RBPMS	1:500	Novus biologicals	NBP2-20112
Mouse monoclonal anti-β-III-tubulin	1:400	Novus biologicals	MAB1195
Mouse monoclonal anti-β3-AR	1:400	Santa cruz biotech	sc-1473
Rabbit monoclonal anti-Iba1	1:500	Abcam	ab178846

Retinal whole mounts and microdissected astrocytes were incubated for 48 h at 4 °C with Alexa Fluor 555-conjugated anti-rabbit secondary antibody (ab150078; Abcam, Cambridge, UK) or Alexa Fluor 488-conjugated anti-rabbit secondary antibody (ab150077; Abcam) both diluted 1:200 in PBS containing 2% Triton X-100. After rinsing in PBS, retinal whole mounts were flat mounted on polarized glass slides with the GCL up coverslipped with Fluoroshield mounting medium. Images were acquired with a 20x Plan-Apochromat objective using an epifluorescence microscope (Ni-E; Nikon-Europe) equipped with a digital camera (DS-Fi1c camera; Nikon-Europe). In the case of glial fibrillary acidic protein-immunoreactivity (GFAP-IR), Z-stack digital images were collected in the central area at every 1 μm depth within 25 or 100 μm from the GCL focal plane for astrocytes or Müller cells, respectively. ImageJ software was used to perform convolution filtering to realize both Z-stack projections and 3D reconstructions. In order to obtain whole-mount images showing astrocytes and Müller cells, Z-stacks were converted into 2D images through the “maximum intensity projection” function from ImageJ. RNA-binding protein with multiple splicing (RBPMS)-IR, paired box 2 (PAX2)-IR and β-III-tubulin-IR to identify RGCs, astrocytes and RGC axons, respectively, were analyzed by confocal microscopy. In the case of RBPMS-IR localization to retinal whole-mounts, RGCs were counted in 4 radial areas in the central avascular zone (210 × 210 μm) at a similar distance from the optic nerve head in order to compare their density in the different experimental groups. In the case of PAX2-IR, immunostained cells were counted in 4 radial areas in both the central avascular zone (500 × 500 μm) and the midperiphery (500 × 500 μm) at a similar distance from the optic nerve head in order to compare their density in the different experimental groups. Quantification of immunostained cell density was performed in a masked manner by measuring the average number of immunopositive somata per mm^2^.

Microdissected astrocytes were coverslipped with Fluoroshield Mounting Medium containing 4′,6-diamidino-2-phenylindole (DAPI, Abcam). Confocal microscopy was used to validate the pull-off technique by verifying GFAP-IR localization to astrocytes. RBPMS-IR and Ionized calcium-binding adaptor molecule 1 (IBA-1)-IR were also evaluated to exclude the presence of RGCs and microglial cells. IB4 staining was also performed to identify retinal vessels. Confocal microscopy was also used to evaluate the specific expression of β3-AR within GFAP-positive astrocytes. Confocal optical sections were obtained by scanning acquisition with a 10x objective and a detector resolution of 1,024 × 1,024 pixels. In some experiments, images were then turned into grayscale, normalized for the background and analyzed for the mean gray levels to quantify the immunofluorescence intensity for each marker in 4 preparations of pull-off isolated astrocytes per experimental group using the analysis tool of Adobe Photoshop.

### 2.7 Quantitative real time RT-PCR

In both whole retinas and microdissected astrocytes, total RNA was purified using a column-based kit (RNeasy Mini Kit; Qiagen, Valencia, CA, USA) and quantified by spectrophotometric analysis (BioSpectrometer basic; Eppendorf AG, Hamburg, Germany). Of the total RNA extracted by whole retinas and microdissed astrocytes, 1 μg and 150 ng, respectively were used to synthesize the corresponding first-strand cDNA using the QuantiTect Reverse Transcription Kit (Qiagen). Real-time PCR amplifications were performed using the SsoAdvanced Universal SYBR Green Supermix (Bio-Rad Laboratories) on a CFX Connect Real-Time PCR detection system equipped with the software CFX Manager (Bio-Rad Laboratories). Forward and reverse primers were chosen to hybridize to unique regions of the appropriate gene sequence with an amplification efficiency close to 100%. Primer sequences are reported in Table 3. The target gene was run concurrently with Rpl13a, a constitutively expressed housekeeping gene encoding the ribosomal protein L13A. Samples were compared using the relative threshold cycle (Ct method) and expressed as fold increase. All reactions were run in triplicate. For each experimental condition, 6 independent samples, each from 2 retinas of 2 different mice were used.

**Table 3:** Primer sequences used for quantitative real time RT-PCR.

Gene	Forward 5^′^-3^′^	Reverse 5^′^-3^′^
β3-AR	TCTCTGGCTTTGTGGTCGGA	GTTGGTTATGGTCTGTAGTCTCG
Rpl13a	CCAGGTATACAAGCAGGTGTGCTC	CATCATTAGGGCCATCCTGGAC
GFAP	AGGGAGTGGAGGAGTCATTCG	CGGAGACGCATCACCTCT
VEGF	GCACATAGGAGAGATGAGCTTCC	CTCCGCTCTGAACAAGGCT
FGF2	GCGACCCACACGTCAAACTA	TCCCTTGATAGACACAACTCCTC
Fibronectin 1	CCCTATCTCTGATACCGTTGTCC	TGCCGCAACTACTGTGATTCGG
Laminin α1	TGTCTGCAAGCCAGGAGCTACA	GAGACAGAACGGCATCACCAAC
Laminin γ1	CTGTAATGGGCACAGTGAGACC	ACAAGGCTGGCAGTCAGAGGAG
Integrin αV	GTGTGAGGAACTGGTCGCCTAT	CCGTTCTCTGGTCCAACCGATA
Integrin β1	ATGCCAAATCTTGCGGAGAAT	TTTGCTGCGATTGGTGACATT
Integrin β8	ATGCTTCAGGCTGCCGTCTGTG	CAGTTTCCGTCATTCGGCACCA
CTNF	AGCAAGGAAGATTCGTTCAGACC	TCTGCCTCAGTCATCTCACTCC
BDNF	TTGTTTTGTGCCGTTTACCA	GGTAAGAGAGCCAGCCACTG

### 2.8 Analysis of BRB leakage using Evans blue dye

For inner BRB assessment, 3 mice from each experimental group were anesthetized by intraperitoneal injection of avertin. Mice underwent systemic perfusion with 0.5% Evans blue dye (Sigma-Aldrich) in PBS, which was intracardially injected into the left ventricle for 10 min. Then, the mice were euthanized and the explanted retinas were flat-mounted onto glass slides. The retinas were examined using an epifluorescence microscope (Ni-E; Nikon-Europe) equipped with a 10x Plan-Apochromat objective and a digital camera (DS-Fi1c camera; Nikon-Europe).

### 2.9 Elettroretinographic recordings

Electroretinography was performed in 6 mice of the 129S strain randomly chosen from the experimental groups. The effect of BRL37344 was analyzed at PD17 with scotopic full-field electroretinogram (ERG), which provides an assessment of general retinal function, and pattern ERG (PERG), a tool extensively used to assess RGC activity. Mice were dark-adapted overnight and anesthetized by an intraperitoneal injection of avertin (1.2% tribromoethanol and 2.4% amylene hydrate in distilled water, 0.02 mL/g body weight; Sigma-Aldrich) and placed on a heating pad to maintain a constant body temperature of 38 °C. ERG recording was performed using an available setup (Retimax Advanced; CSO, Firenze, Italy). To maintain corneal moisture balanced saline solution was instilled throughout the ERG session. ERG responses were recorded using Ag/AgCl corneal electrodes. A reference electrode was placed subcutaneously behind the ear, and a ground electrode was connected to the base of the tail. Scotopic full-field ERG responses were evoked by flashes of increasing light intensities (from −3.4 to 1 log cd-s/m^2^) delivered via a Ganzfeld bowl. After 5,000-fold amplification and 1–100 Hz band-pass filtering, the scotopic ERG waveforms were analyzed to obtain the amplitudes of the a-wave (from the pre-stimulus baseline to the negative trough) and b-wave (from the trough of the a-wave to the following positive peak). For PERG recording, the light emitting diode display was aligned at about 25 cm from the corneal surface and used to deliver pattern reversal stimuli consisting of black and white horizontal bars (spatial frequency: 0.05 cycles/deg; temporal frequency: 1 Hz; contrast: 98%) with a mean luminance of 50 cd/m^2^. After averaging a total of 300 consecutive responses to reduce noise contamination, PERG waveforms were analyzed by detecting the early negative inflection point (N1), the positive peak (P1) and the belated negative trough (N2) to measure N1-P1 and P1-N2 amplitudes.

### 2.10 Statistical analysis

Statistical analyses were performed using Prism 8.0.2 software (GraphPad Software, Inc., San Diego, CA, United States). The normal distribution of the data was verified using the Shapiro-Wilk test. Unpaired Student's *t*-test, One-way ANOVA followed by Tukey's *post-hoc* test or two-way ANOVA followed by Bonferroni's *post-hoc* test, were used to analyze the differences between experimental groups, as appropriate. Differences with *P* < 0.05 were considered significant. Data are expressed as box-and-whisker plots, with boxes representing the 25^th^-75^th^ percentiles and whiskers indicating the minimum and maximum values, with individual data points.

## 3 Results

### 3.1 β3-AR agonism prevents neovascular response to hypoxia

In the 129S strain, the OIR-associated vascular response was investigated by IB4 staining with respect to that of the C57 strain. In the 129S strain, the vascular pattern in response to hypoxia differs substantially from that in the C57 strain with a tuft area that was about 160% higher than that in the C57 strain. The avascular area did not differ between the two strains. The higher susceptibility of 129S to hypoxia was paralleled by a drastic upregulation of β3-AR, as demonstrated by increased levels of β3-AR protein that were approximately 350% higher than those measured in OIR mice of the C57 strain (Supplementary Figure 1).

**Figure 1:**
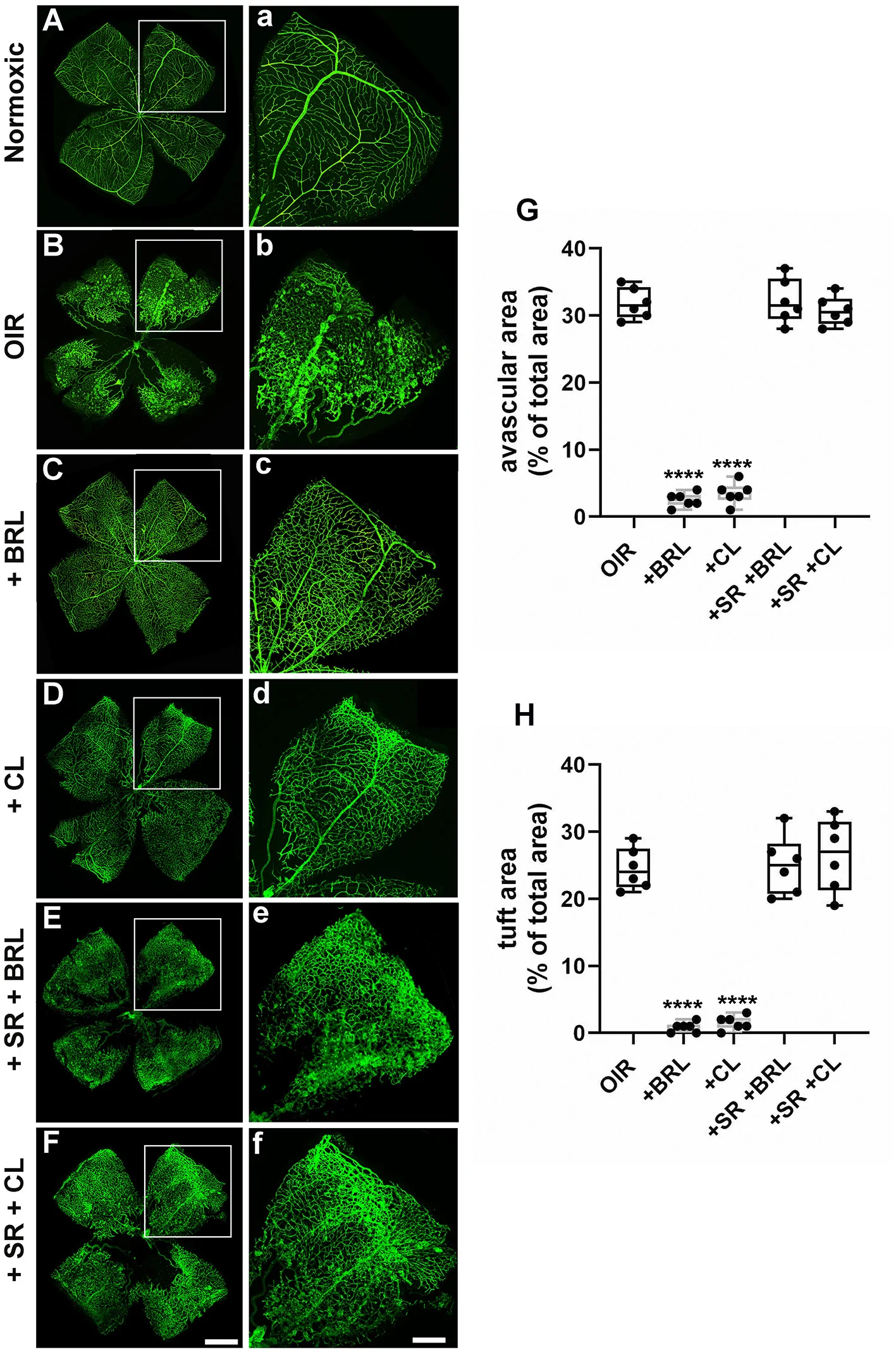
Either BRL37344 or CL316243 promotes central retina revascularization and reduces the neovascular tuft area. **(A–F)** Representative images of IB4 labeled superficial plexus of retinas from PD17 mice normoxic, OIR untreated or treated with BRL37344 (BRL) or CL316243 (CL) either alone or in combination with the β3-AR antagonist SR59230A (SR) (scale bar: 1 mm). **(a–f)** High magnification of the boxed areas in **A**-**F** (scale bar: 500 μm). The extent of both the avascular area **(G)** and the tuft area **(H)** was quantitatively evaluated in OIR mice either untreated or treated as in the representative images. Data are shown as box plots with minimum to maximum whiskers with individual data points (*n* = 6 retinas). ****P < 0.0001 vs. OIR.

In the 129S mouse strain, the effect of β3-AR agonism on OIR-associated vascular pattern was investigated using vessel staining with IB4. Figure 1 shows the vascular pattern of retina flatmounts in normoxic and OIR mice untreated or treated with BRL37344 or CL316243. Both agonists were administered either alone or in combination with the β3-AR antagonist SR59230A. With respect to normoxic mice, untreated OIR mice displayed a pronounced central avascular area and tumultuous neovessel formations extending between the obliterated central region and the peripheral retina. The administration of either BRL37344 or CL316243 was found to promote vessel regrowth into the central avascular area and to prevent the development of neovascular tufts in the midperiphery. The finding that two distinct agonists produce the same response supports the functional involvement of the receptor. Preventive administration of the β3-AR antagonist SR59230A was found to abrogate the ameliorative effect of β3-AR agonism. Quantitative analysis revealed that both agonists significantly reduced the avascular area (by about 92 and 89%, respectively) and much less tuft formation in the midperiphery (by about 96 and 91%, respectively) with respect to the retinas of untreated mice. As also shown quantitatively, both effects were abrogated by SR59230A.

Because BRL37344 and CL316243 produced overlapping protective effects, using both agonists in further experiments aimed at investigating the β3-AR role in the retinal recovery from hypoxic damage would represent pharmacological redundancy rather than mechanicistic validation. For this reason, subsequent experiments employed BRL37344 as a well-characterized agonist for its broader efficacy in preclinical studies (Cernecka et al., 2014).

**Figure 2:**
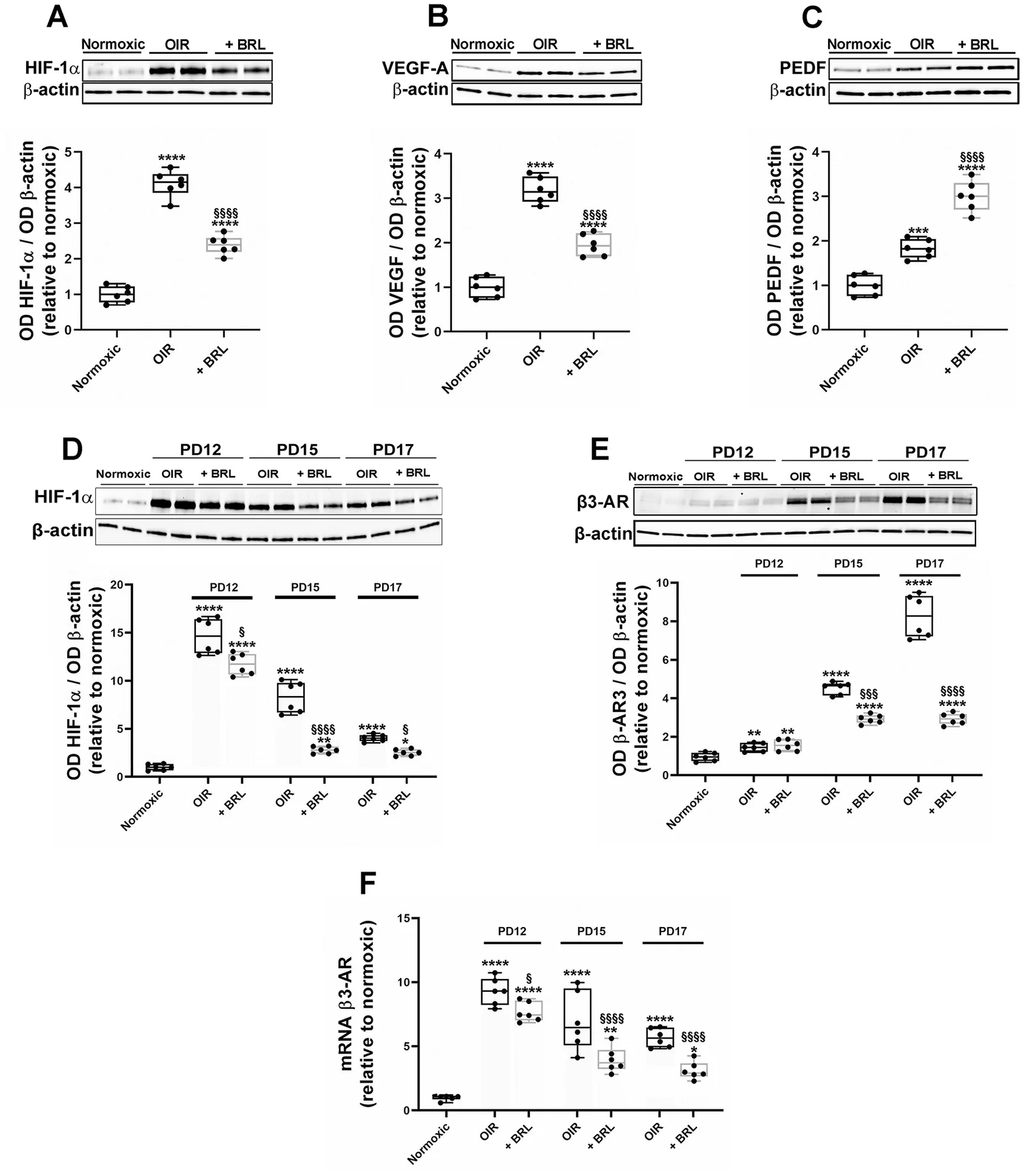
BRL37344 affects hypoxia-driven angiogenic markers and restrains the upregulation of HIF-1α and β3-AR during the hypoxic phase. **(A–C)** Representative Western blots and relative densitometric analysis of the α subunit of HIF-1 (HIF-1α), vascular endothelial growth factor (VEGF), and pigment epithelium-derived factor (PEDF) protein levels in retinal homogenates from PD17 mice, normoxic, OIR untreated or treated with BRL37344 (BRL). **(D, E)** Representative Western blots and relative densitometric analysis of HIF-1α and β3-AR protein levels in normoxic and OIR mice either untreated or treated with BRL37344 and sampled at PD12, PD15 and PD17 after BRL37344 (BRL) was administered starting from 6 h after returning to normoxia. **(F)** Levels of β3-AR mRNA as determined by qPCR. In Western blot analysis, β-actin was used as a loading control. Data are shown as box plots with minimum to maximum whiskers with individual data points (*n* = 6 samples). *P < 0.05; **P < 0.01; ***P < 0.001; ****P < 0.0001 vs. normoxic; ^§^*P* < 0.05; ^§§§^*P* < 0.001; ^§§§§^*P* < 0.0001 vs. OIR.

As shown in Figures 2A–C, the beneficial effect of BRL37344 on hyperoxia-associated vaso-obliteration of the central retina prevents the onset of hypoxia leading to a reduced angiogenic stimulus during the second phase of OIR, as evidenced by decreased levels of both HIF-1α and VEGF, and increased levels of the anti-angiogenic factor pigment epithelium-derived factor (PEDF) in retinal homogenates. In particular, in the OIR retina, the levels of pro-angiogenic markers HIF-1α and VEGF were higher than in normoxic controls (about 300% and 200%, respectively). After the administration of BRL37344, both HIF-1α and VEGF levels decreased by about 41% compared to untreated OIR mice, although they remained significantly higher than in normoxic controls. In OIR mice, the retinal levels of PEDF increased by about 80% with respect to normoxic controls, and an additional 70% increase was observed following BRL37344 administration compared with untreated OIR. As also shown in Figures 2D–F, HIF-1α downregulation after BRL37344 was found to affect the expression of β3-AR in response to hypoxia in line with the finding that HIF-1α transactivates the β3-AR gene (Amato et al., 2022). By correlating the trend of HIF-1α expression with that of β3-AR at different times after the onset of hypoxia, we found that at PD12 (6 h after the beginning of hypoxia), HIF-1α protein was maximally upregulated but then progressively decreased until PD17, when its level was approximately 72% lower than at PD12 but still about 300% higher than in normoxic mice. Upregulation of β3-AR protein became evident at PD15, when its levels were about 200% higher than at PD12 but then increased drastically at PD17 (about 80% higher than at PD15). Except for β3-AR protein at PD12, at each time point BRL37344 was found to significantly prevent upregulated levels of HIF-1α and β3-AR with progressively increasing efficacy until PD15. Concomitant variations of β3-AR messenger are indicative of agonism efficacy at the level of gene expression. In fact, β3-AR messenger progressively decreased from PD12 to PD17, when its expression was about 41% lower than at PD12.

### 3.2 β3-AR agonism prevents BRB breakdown and leads to restored retinal function

**Figure 3:**
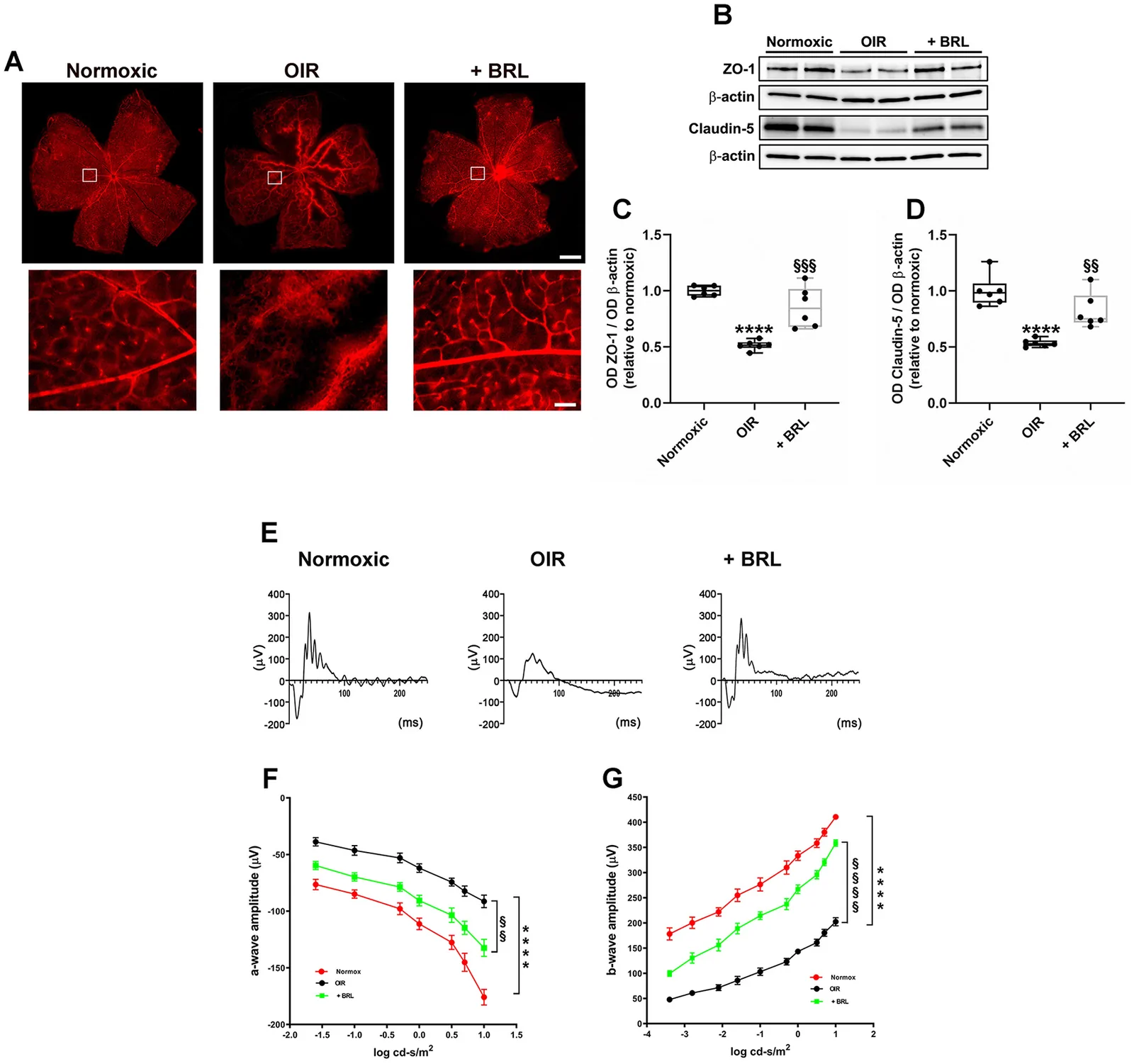
BRL37344 prevents blood-retinal barrier breakdown and electroretinogram (ERG) dysfunction. **(A)** BRB leakage was qualitatively evaluated with the Evans blue dye in PD17 mice normoxic, OIR untreated or treated with BRL37344 (BRL). Six retinas were used for each experimental condition. Scale bar: 1 mm in the low magnifications and 500 μm in the high magnifications. **(B)** Representative Western blots of zonula occludens (ZO)-1 and claudin-5 protein levels in retinal homogenates from PD17 mice either normoxic, OIR untreated or treated with BRL37344 (BRL). Their relative densitometric analysis is shown in **C** and **D**. β-actin was used as a loading control. Data are shown as box plots with minimum to maximum whiskers with individual data points (*n* = 6 samples). ****P < 0.0001 vs. normoxic; ^§§^*P* < 0.01; ^§§§^*P* < 0.001 vs. OIR. **(E)** Representative ERG waveforms recorded at a light intensity of 1 log cd-s/m^2^ in PD17 mice either normoxic or OIR untreated or treated with BRL37344 (BRL). Amplitudes of the scotopic a-wave **(F)** and b-wave **(G)** are plotted as a function of increasing light intensity in normoxic (red circles and red line) and OIR mice untreated (black circles and black line) or treated with BRL37344 (BRL; green squares and green line). Each point represents the mean ± SEM of data from 6 mice. ****P < 0.0001 vs. normoxic; ^§§^*P* < 0.01; ^§§§§^*P* < 0.0001 vs. OIR.

To evaluate whether the ameliorative effects of BRL37344 on HIF-1α and VEGF levels were reflected in BRB recovery, the effect of BRL37344 on OIR-associated breakdown of the inner BRB was investigated by determining the extravasation of Evans blue dye. In addition, the protein levels of zonula occludens 1 (ZO-1), a cytosolic scaffold that regulates the assembly of cellular junctions and claudin-5, a transmembrane protein that plays a key role in the paracellular barrier to small molecules were also determined. Loss of ZO-1 and claudin-5 is a central event in determining BRB dysfunction (Greene et al., 2019; Bora et al., 2023). The possibility that recovered BRB breakdown might be related to restored retinal dysfunction was investigated by determining the amplitudes of scotopic ERG waveforms in OIR mice either untreated or treated with BRL37344 as compared with normoxic mice. As shown in Figures 3A–D, OIR-associated extravasation of Evans blue was accompanied by about 50% decreased levels of ZO-1 and claudin-5 in untreated OIR mice. After BRL37344, the inner BRB integrity was recovered and the protein levels of ZO-1 and claudin-5 were almost comparable to those determined in normoxic controls. As shown in Figures 3E–G, OIR mice showed a reduction in the amplitudes of scotopic a- and b-waves at all light intensities, reaching an almost 50% decrease at the highest stimulus intensity. After BRL37344, the a- and b-wave amplitudes were significantly higher than in untreated mice but still lower than in normoxic mice.

### 3.3 β3-AR agonism restores the astrocyte template

**Figure 4:**
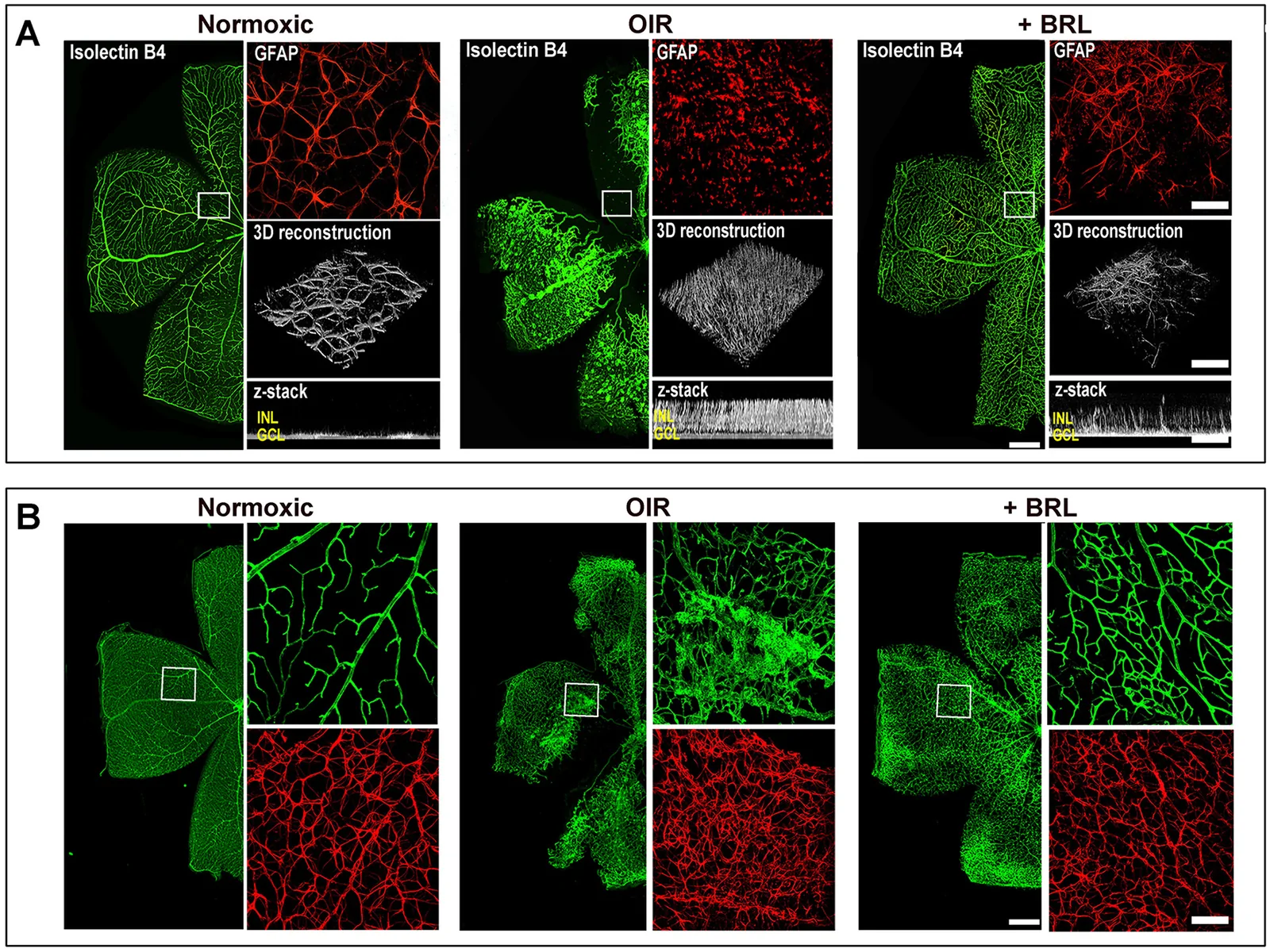
BRL37344 prevents astrocyte loss in the central retina and reduces astrocyte mispatterning in the midperiphery in concomitance with recovered vascularization. **(A)** Representative images of whole-mount retinas stained with IB4 (in green) from PD17 mice normoxic, OIR untreated or treated with BRL37344 (BRL) (low magnification in the left panel, Scale bar: 1 mm). Boxed areas correspond to high-magnification images of the central retina showing GFAP-IR (in red). Scale bars: 50 μm. 3D reconstructions and Z-stack projections of GFAP-IR display astrocyte profiles and reactive Müller cell processes, respectively. Scale bars: 50 μm in 3D reconstructions and 70 μm in Z-stack projections. **(B)** Representative images of whole-mount retinas stained with IB4 (in green) from PD17 mice normoxic, OIR untreated or treated with BRL37344 (BRL) (low magnification in the left panel, Scale bar: 1 mm). Boxed areas correspond to high-magnification images of the midperipheral retina showing GFAP-IR (in red) and IB4 stained vessels (in green). Six retinas were used for each experimental condition Scale bar: 50 μm.

Whether restored vessel integrity following BRL37344 is correlated with astrocyte recovery was investigated in both the retinal center and the midperiphery by analyzing GFAP-IR with respect to IB4 staining of retinal vessels (Figure 4). In Figure 4A, the central retina of normoxic controls was characterized by regularly distributed astrocytes with well-organized branching profiles as evidenced by GFAP-IR (in red) in concomitance with IB4 stained vessels (in green). Instead, the central avascular retina of untreated OIR mice was characterized by astrocyte loss as demonstrated by GFAP-IR, which failed to delineate astrocyte profiles but rather appeared to be localized to gliotic Müller cells. In addition to restoring the vascularization of the central retina, BRL37344 was found to recover well-defined GFAP-positive astrocyte profiles. 3D reconstructions and Z-stack projections of the central retina demonstrated that under normoxia no GFAP-IR could be attributed to Müller cells as shown by the negligible presence of GFAP-positive orthogonal processes. In OIR, dense GFAP-immunostained profiles indicative of reactive Müller cells could be observed while astrocytes could not be visualized by GFAP-IR. Müller cell gliosis drastically decreased after BRL37344 when astrocyte profiles were almost recovered. In Figure 4B, the midperipheral retina of normoxic controls was characterized by well delineated astrocyte profiles that followed the flow of the retinal vessels, which in OIR displayed engorged tufts accompanied by intricate astrocyte assembly. BRL37344 administration was found to prevent mispatterning of both vessels and astrocytes, which recovered their normal distribution and morphology.

**Figure 5:**
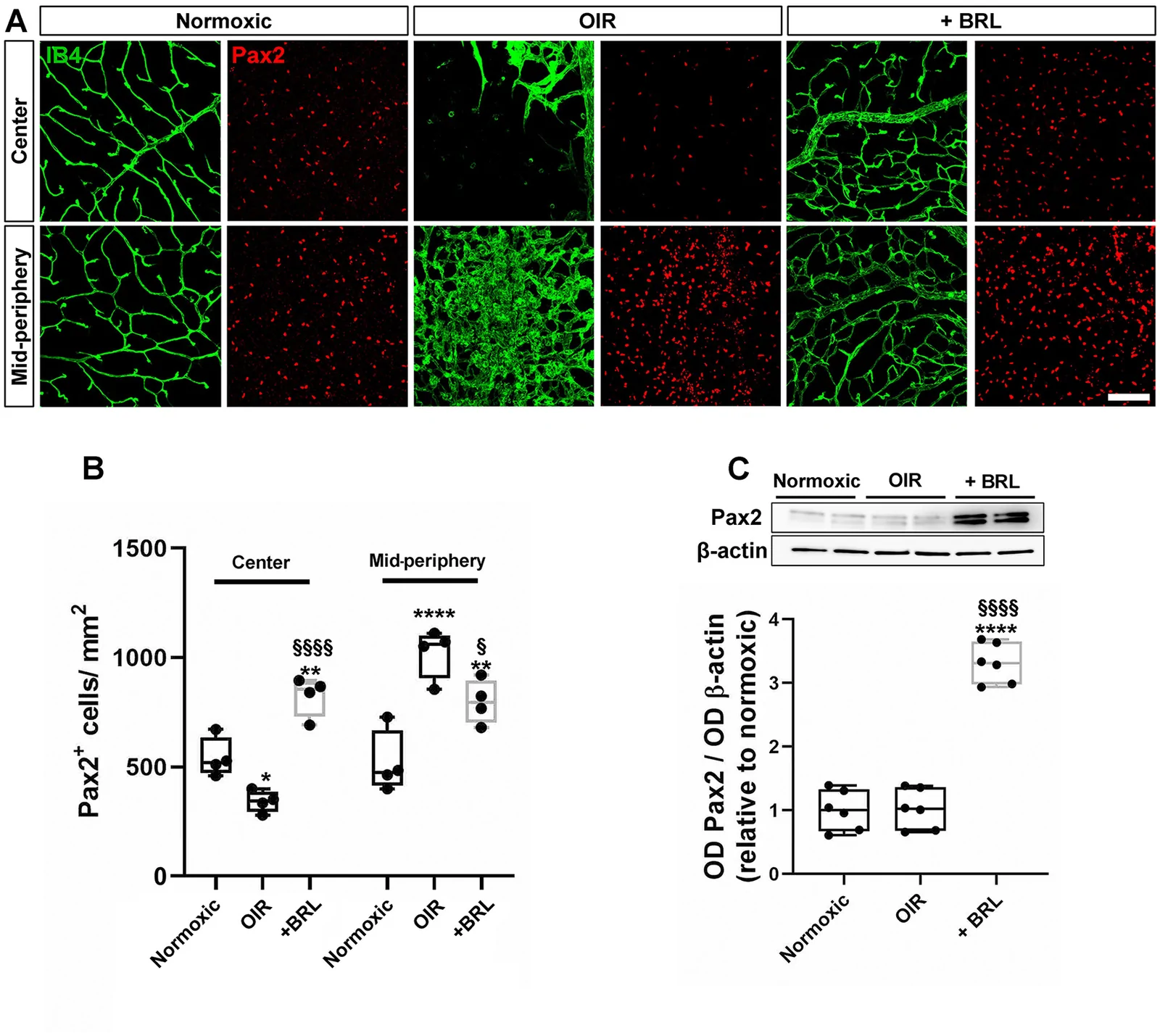
BRL37344 prevents altered expression of PAX2-IR both in the center and the midperiphery. **(A)** In the central retina of OIR mice, the reduced density of PAX2 immunoreactive astrocytes (in red) is concomitant with the loss of IB4 stained vessels (in green), while the increased density of astrocytes in the midperiphery is concomitant with dysregulated vessel formation. Both vessels and astrocytes appear to recover their normal pattern after BRL37344 (BRL) although cell count reveals a persistently increased number of PAX2 positive cells both in the center and the midperiphery. **(B)** Four retinas were used for each experimental condition. Scale bar: 150 μm. **(C)** Representative Western blots and relative densitometric analysis of PAX2 protein levels in retinal homogenates from PD17 mice normoxic, OIR untreated or treated with BRL37344 (BRL). β-actin was used as a loading control. Data are shown as box plots with minimum to maximum whiskers with individual data points (*n* = 4/6 samples). *P < 0.05, **P < 0.01; ****P < 0.0001 vs. normoxic; ^§^*P* < 0.05; ^§§§§^*P* < 0.0001 vs. OIR.

Whether β3-AR agonism promotes the recovery of the astrocyte template in both the center and the midperiphery was investigated by analyzing the immunostaining pattern of PAX2, a transcription factor involved in astrocyte migration and maturation (Soukkarieh et al., 2007; Tao and Zhang, 2014). As shown in Figure 5A, in the central retina, vessel loss in response to OIR was accompanied by astrocyte loss, which was prevented by BRL37344 leading to a massive astrocyte density exceeding that in control conditions. In the midperiphery, OIR led to an increased density of PAX2 positive astrocytes with altered spatial patterning concomitant with the occurrence of dysregulated vessel formation, an event that was attenuated by BRL37344. In OIR, quantitative analysis of PAX2-IR revealed that the number of PAX2 positive cells in the central retina decreased by about 33% with respect to the control condition while a 100% higher number was found in the midperiphery. The PAX2-IR pattern after BRL37344 failed to regain control conditions with cell numbers exceeding those in the control by about 50% both in the center and the midperiphery (Figure 5B). As shown in Figure 5C, Western blot analysis of PAX2 proteins in retinal homogenates revealed no significant difference between normoxic and OIR conditions presumably because the increased number of PAX2-positive cells in the midperiphery compensates for astrocyte loss in the center. Increased PAX2 levels by about 220% after BRL37344 with respect to untreated OIR might reflect the persistently increased number of PAX2 positive cells both in the center and the midperiphery.

### 3.4 β3-AR agonism promotes RGC rescue in the central retina

**Figure 6:**
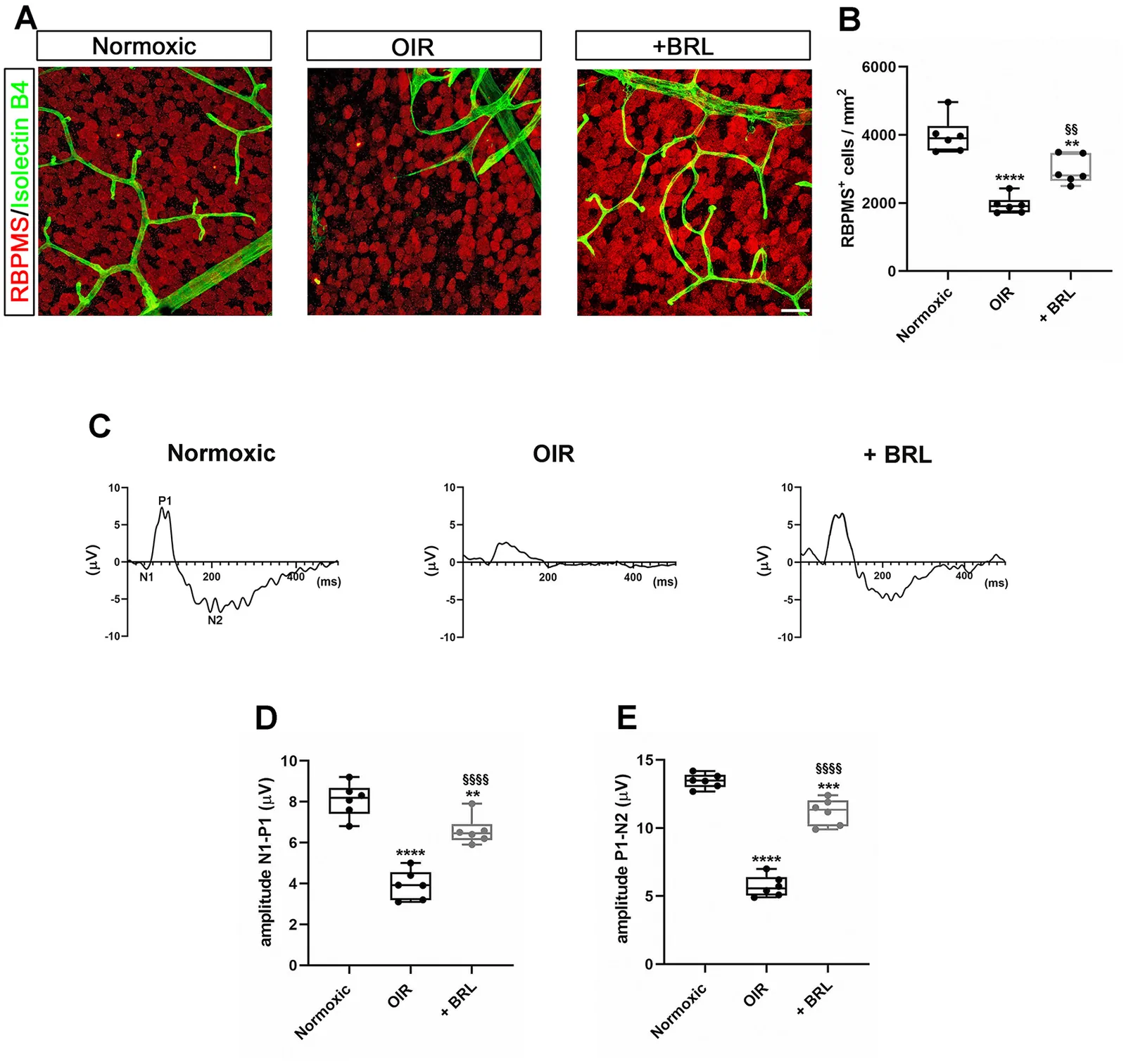
BRL37344 prevents OIR-associated RGC loss and PERG alterations. **(A)** Representative images of RBPMS-labeled RGCs (in red) in relation to IB4 staining of vessels (in green) in the central area of whole-mount retinas from PD17 mice normoxic, OIR untreated or treated with BRL37344 (BRL). Scale bar: 25 μm. **(B)** Quantitative evaluation of RBPMS-labeled cell density. **(C)** Representative PERG waveforms and quantitative analysis of N1-P1 and P1-N2 amplitudes. **(D, E)** Data are shown as box plots with minimum to maximum whiskers with individual data points (*n* = 6 mice). **P < 0.01; ***P < 0.001; ****P < 0.0001 vs. normoxic; ^§§^*P* < 0.01; ^§§§§^*P* < 0.0001 vs. OIR.

Drastic loss of RGCs characterizes the retina of OIR mice in which we verified whether recovered vascularization by β3-AR activation might preserve RGC viability. As shown in Figure 6, we determined RGC density using immunostaining for RBPMS, a selective marker of RGCs (Rodriguez et al., 2014) to then evaluate RGC function by PERG recordings that are indicative of RGC activity (Porciatti et al., 2007). In the retina of OIR mice, RGC loss was evident in concomitance with the decreased amplitude of the N1-P1 and P1-N2 waves that correspond to the activity of the central retina. OIR mice treated with BRL37344 displayed higher RGC density in comparison with untreated mice together with the recovered amplitude of the PERG waveforms. As shown by the quantitative analysis, the number of RBPMS positive cells decreased by about 50% after hypoxia to then increase by about 25% after BRL37344. Concomitantly, the amplitude of the N1-P1 and P1-N2 waves was reduced by approximately 44% compared to normoxic mice. After BRL37344, the amplitude of the N1-P1 and P1-N2 waves increased by about 70% and 100%, respectively while remaining about 20% lower than in normoxic mice.

**Figure 7:**
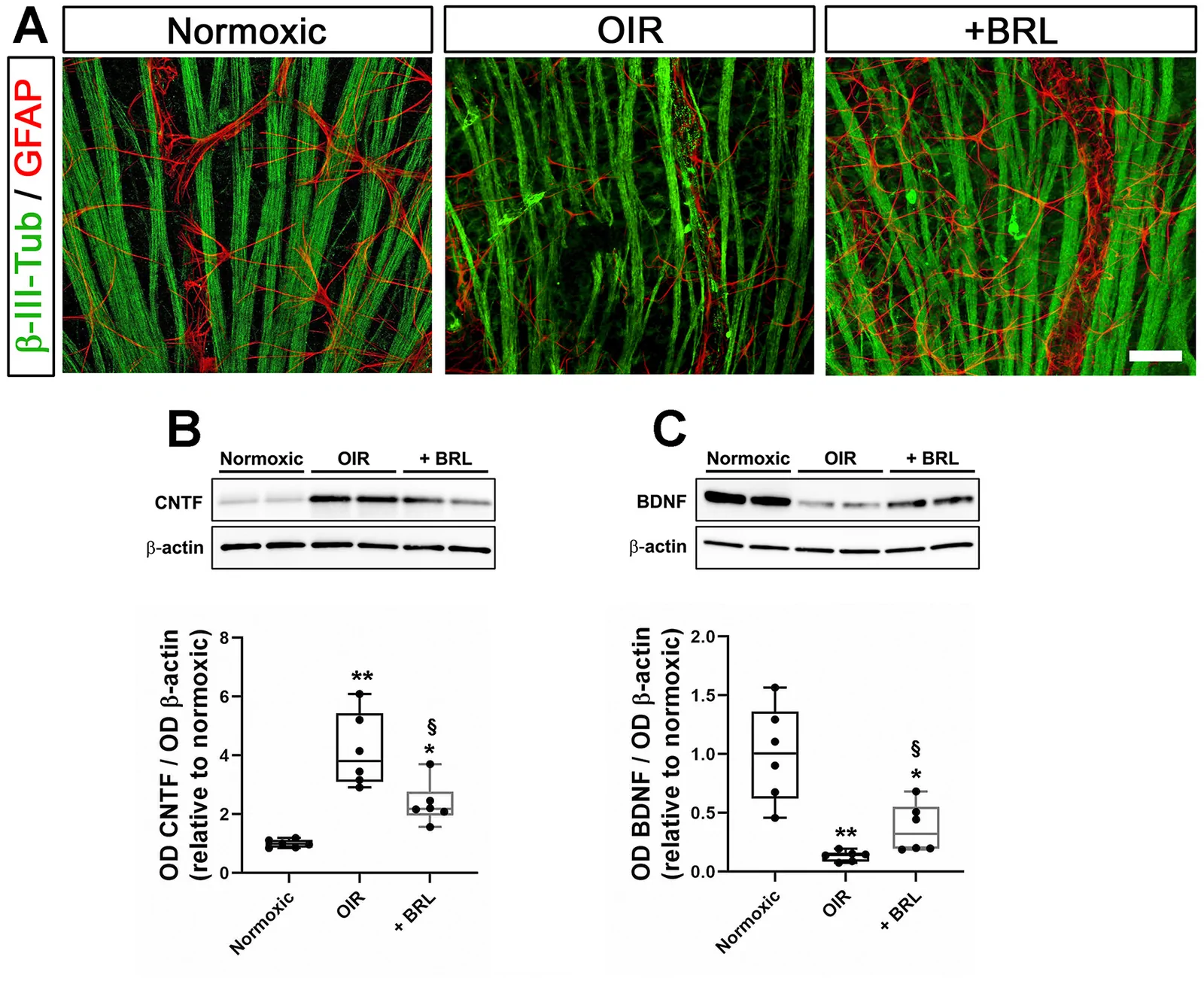
BRL37344 prevents OIR-associated loss of both RGC axons and astrocytes and counteracts alterations in CNTF and BDNF levels. **(A)** Representative images of β-III-tubulin labeled RGC axons (green) and GFAP labeled astrocytes (red) in PD17 mice normoxic, OIR untreated or treated with BRL37344 (BRL). Six retinas were used for each experimental condition Scale bar: 50 μm. **(B, C)** representative Western blots and relative densitometric analysis of protein levels of CNTF and BDNF in retinal homogenates from PD17 mice normoxic, OIR untreated or treated with BRL37344 (BRL). β-actin was used as loading control. Data are shown as box plots with minimum to maximum whiskers with individual data points (*n* = 6 samples). *P < 0.05; **P < 0.01 vs. normoxic; ^§^*P* < 0.05 vs. OIR.

Whether the recovered density of RGCs might be potentially correlated with the restored activity of astrocytes, which provide them with extensive support within the retina and along RGC axons was also determined by immunostaining of the central retina with β-III-tubulin to label RGC axons (Jiang et al., 2015) in combination with GFAP to label astrocytes. β-III-tubulin is a protein component of microtubules already used to identify and quantify RGCs with their axons in models of optic nerve injury and glaucoma. Whether the preservation of RGC viability by BRL37344 could coincide with restored levels of neurotrophic factors that are known to mediate RGC survival (Abraham and Telias, 2025) was also investigated. As shown in Figure 7A, with respect to normoxic controls, in OIR, β-III-tubulin expression decreased in line with RGC loss. GFAP immunolabeled astrocytes, which are interspersed in the nerve fiber layer, were concomitantly reduced to almost regain their control density after BRL37344 together with recovered β-III-tubulin-IR. As shown in Figures 7B, C in concomitance with restored RGC axons and astrocytes, BRL37344 prevents OIR-associated alterations in the levels of neurotrophic factors by reducing upregulated levels of the ciliary neurotrophic factor (CNTF) while increasing downregulated levels of the brain-derived neurotrophic factor (BDNF). In untreated OIR, CNTF levels were increased by about 300%, then becoming almost halved after BRL37344 presumably as a consequence of attenuated astrocyte activation. Levels of BDNF were decreased by about 87% possibly related to RGC loss. After BRL37344, BDNF levels almost tripled potentially in relation to RGC resiliency.

### 3.5 β3-AR coupling to recovered astrocytes in microdissected preparations

In light of the important contribution of astrocytes to retinal angiogenesis, simplified models of astrocytes microdissected from the retinas of normoxic mice, OIR untreated or treated with BRL37344 were used to investigate whether BRL37344 might directly affect angiogenesis-related factors such as VEGF and fibroblast growth factor 2 (FGF2), potentially through β3-AR localized to the astrocyte membrane. Whether β3-AR agonism might affect the levels of fibronectin, a key extracellular matrix (ECM) component expressed by astrocytes, laminins, the core components of the basement membrane, and integrins, the major receptors used by astrocytes to bind laminins, was also investigated.

Despite the advantage of the model of microdissected astrocytes which allows us to overcome the difficulty of their low number (about 0.1% of the retinal cells), their sparse distribution and similarity to more abundant Müller cells (Cullen et al., 2023), the scarce availability of the biological material has limited the use of the preparation except for mRNA detection.

Validation of the pull-off technique leading to microdissected astrocytes is shown in Supplementary Figure 2 in which GFAP-labeled astrocytes are clearly visible while RGCs, microglia and vessels cannot be evidenced as demonstrated by the lack of their associated IR.

The specific expression and functional presence of β3-AR on retinal astrocytes is considered a fundamental prerequisite for investigating the role of β3-AR in these cells. The specificity of the β3-AR antibody used in the present study was validated by omitting the primary antibody and determining the potential β3-AR labeling in astrocytes isolated from the retinas of β3-AR^−/−^ mice. As shown in Supplementary Figure 3, in both experimental conditions, no β3-AR labeling could be observed.

**Figure 8:**
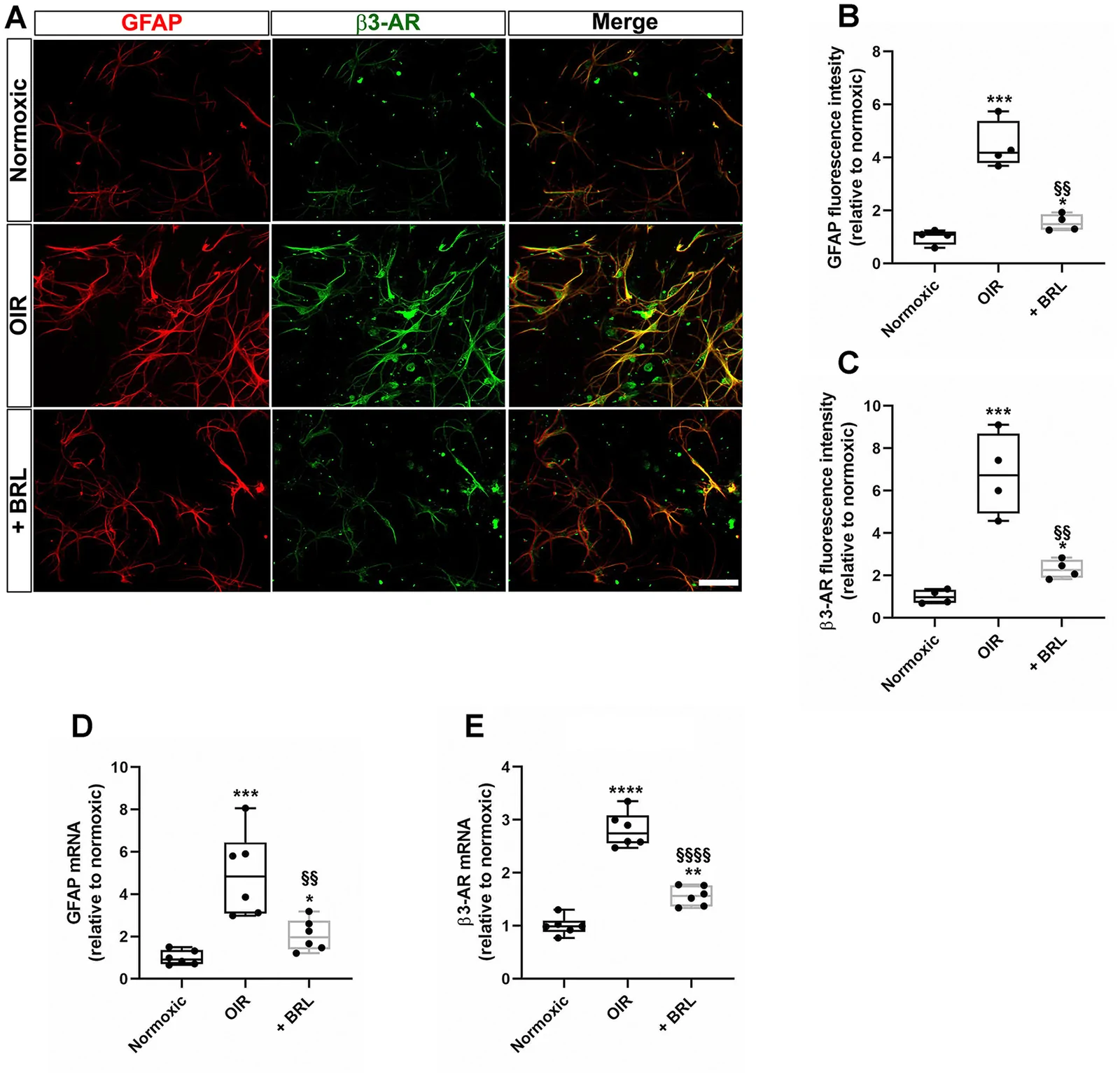
Colocalization of β3-AR-IR and GFAP-IR and their messenger regulation by BRL37344. **(A)** Representative images of microdissected astrocytes immunostained with GFAP and β3-AR from mice either normoxic or OIR, untreated or treated with BRL37344 (BRL). Scale bar: 50μm. **(B, C)** Quantitative analysis of fluorescence intensity. **(D, E)** Levels of GFAP and β3-AR messengers as determined by RT-PCR in as determined by RT-PCR in astrocytes isolated from retinas of mice either normoxic or OIR, untreated or treated with BRL37344 (BRL). Data are shown as box plots with minimum to maximum whiskers with individual data points (*n* = 4/6 samples). *P < 0.05; **P < 0.01; ***P < 0.001; ****P < 0.0001 vs. normoxic; ^§§^*P* < 0.01; ^§§§§^*P* < 0.0001 vs. OIR.

As shown in Figure 8, β3-AR is indeed expressed by isolated astrocytes in which it was colocalized with GFAP, the primary marker of astrocytes. Both β3-AR-IR and GFAP-IR were found to increase in response to hypoxia while they were drastically reduced in astrocytes isolated from the retinas of mice that received BRL37344 (Figure 8A). Quantitative analysis of fluorescence intensity showed that GFAP-IR and β3-AR-IR increased in response to hypoxia by almost 340% and 570%, respectively while they decreased by almost 66% and 65% after BRL37344 administration to OIR mice (Figures 8B, C). The levels of both GFAP and β3-AR messengers confirmed the immunostaining results. In fact, they increased by about 400% and 180%, respectively in response to hypoxia to then decrease by about 58% and 47% after BRL37344 administration (Figures 8D, E).

**Figure 9:**
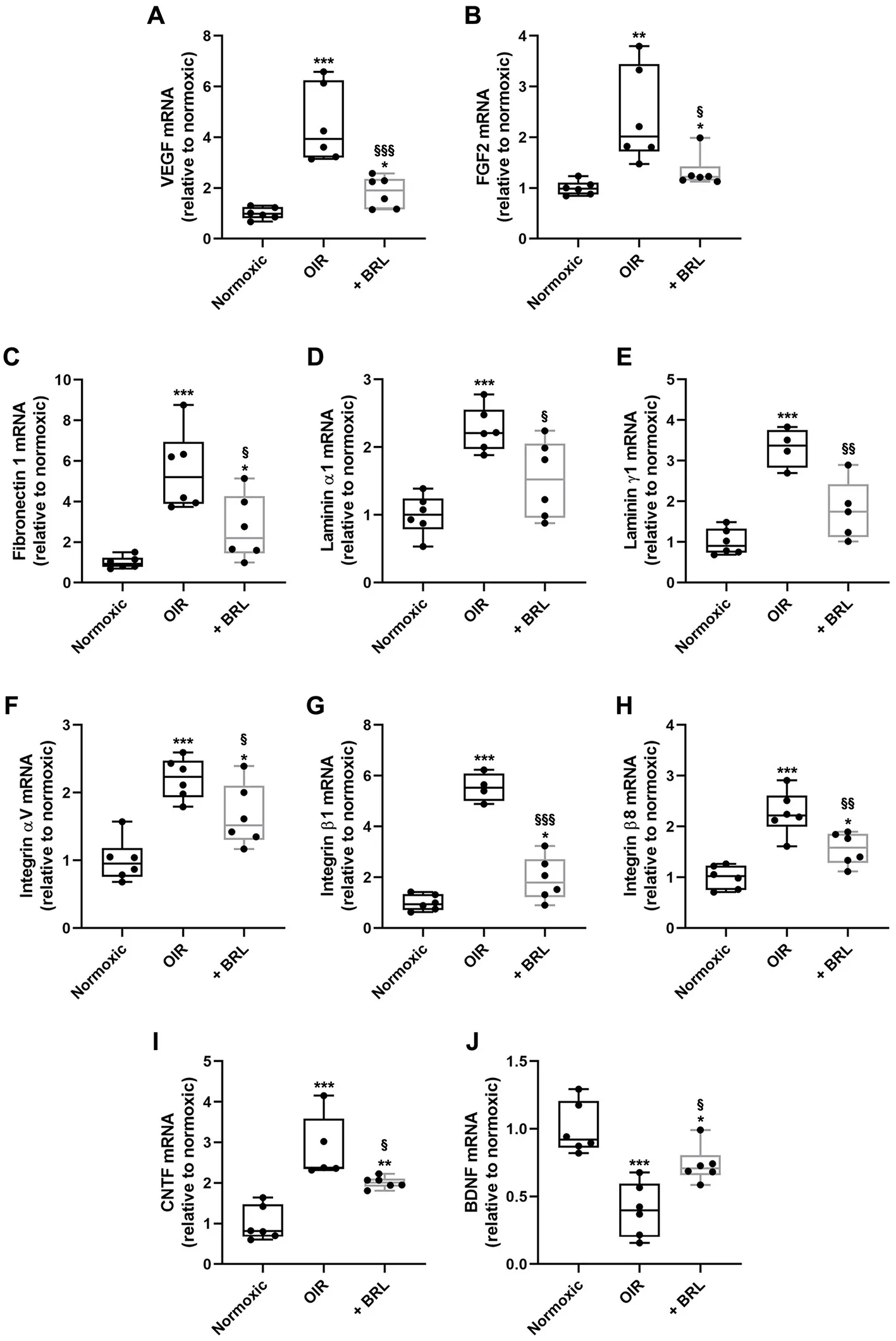
Regulation by BRL37344 of genes related to the angiogenic cascade, ECM components and neurotrophic factors. Relative mRNA levels of VEGF **(A)**, FGF2 **(B)**, fibronectin 1 **(C)**, laminin α1 **(D)**, laminin γ1 **(E)**, integrin αV **(F)**, integrin β1 **(G)**, integrin β8 **(H)**, CNTF **(I)**, and BDNF **(J)** as determined by RT-PCR in astrocytes isolated from retinas of mice either normoxic or OIR, untreated or treated with BRL37344 (BRL). Data are shown as box plots with minimum to maximum whiskers with individual data points (*n* = 5/6 samples). *P < 0.05, **P < 0.01, ***P < 0.001 vs. normoxic; ^§^*P* < 0.05, ^§§^*P* < 0.01, ^§§§^*P* < 0.001 vs. OIR.

In isolated astrocytes, messenger levels of the pro-angiogenic factors VEGF and FGF2, which are secreted by astrocytes in response to hypoxia (Feng et al., 2025; Zhang et al., 2026), were drastically increased in OIR to be then reduced by BRL37344, indicating that β3-AR activation participates in the recovery of the astrocyte-associated pro-angiogenic signaling. As shown in Figures 9A, B, levels of both VEGF and FGF2 were about 350% and 150% higher than in normoxic conditions and then decreased by about 60% and 46% in response to BRL37344.

Fibronectin, laminins and integrins are major components of the basement membrane, providing structural scaffolding and cell adhesion. They are expressed by astrocytes where they are indispensable for their migration and subsequent template formation leading to blood vessel proliferation. In astrocytes from OIR mice, messenger levels of fibronectin 1, laminin α1 and laminin γ1 increased by about 425%, 125% and 220%, respectively. After BRL37344, they decreased by about 50%, 33% and 45%, respectively (Figures 9C–E). Similarly, mRNA levels of integrins αV, β1 and β8 increased by about 120%, 450% and 125%, respectively. After BRL37344, they decreased by about 25%, 65% and 30%, respectively (Figures 9F–H).

In addition to providing the operating factors that mediate the formation of the astrocyte template, astrocytes supply trophic support to neighboring neurons through the local expression of neurotrophic factors including CNTF and BDNF both of which are affected by ischemic conditions (Lee et al., 2022, 2025; Tanase et al., 2025). As shown in Figures 9I, J, in astrocytes isolated from the retinas of OIR mice, messenger levels of CNTF were about 184% higher than in normoxic conditions while BDNF levels were about 60% lower. Both CNTF and BDNF levels were almost restored in astrocytes from the retinas of mice that received BRL37344 with a 30% decrease in CNTF levels and a 75% increase in BDNF levels.

## 4 Discussion

As shown by the present results, β3-AR activation facilitates the revascularization of the central retina in association with recovered astrocyte template thereby allowing the formation of the vascular network that restores the avascular zone. Recovery of the avascular area prevents the development of severe hypoxia and consequently counteracts the neovascular response of the midperipheral retina. Recovered vascularization of the central retina prevents ischemia-associated RGC loss in association with restored astrocytes that support RGC axons by providing them with restored levels of neurotrophic factors. Importantly, activation of β3-AR on astrocytes may represent a regulatory mechanism that mitigates their hypoxia-associated reactivity and regulates their expression of angiogenic markers, ECM components and neurotrophic factors. Overall, these data indicate that β3-AR signaling may serve as an additional mechanism of reprogramming reactive astrocytes toward a more homeostatic and neuroprotective phenotype to stabilize the retinal neurovascular environment.

### 4.1 Strain-dependent OIR response

In terms of OIR, strain-dependent differences have been observed in the angiogenic response to hypoxia. As an example, both the albino BALB/cByJ and the pigmented C57BL/6J strains exhibit vascular regression after hyperoxia but only the C57BL/6J strain develops pathological neovascularization in response to hypoxia while the BALB/cByJ strain restores a normal vascular pattern. Both strains also differ in terms of astrocyte response to hyperoxia with hyperoxia-driven astrocyte degeneration in C57BL/6J mice while in BALB/cByJ mice, astrocytes are unaffected, allowing for physiological vascular regrowth during the hypoxic phase (Dorrell et al., 2010). As shown by the present results, the 129S strain is much more responsive to hypoxia compared to the C57 strain with a higher density of engorged retinal tufts. The response of the 129S strain is characterized by massive β3-AR upregulation and therefore, is suitable for unveiling the role of β3-AR in the vascularization process. In this respect, the potency and efficacy of β3-AR agonists have been found to depend on receptor density on the cell membrane (Wilson et al., 1996) as in models of heart failure in which agonism efficacy requires β3-AR overexpression (Michel et al., 2020) or some predominance over the other subtypes as in the case of β1/2-AR blockade (Trappanese et al., 2015). Whether the observed effect is specific to the 129S strain in which drastic β3-AR upregulation in response to hypoxia promotes agonism effectiveness, the translational potential of β3-AR agonists remains questionable mostly in the absence of any reliable evidence of β3-AR expression in the human retina. In fact, a small number of human tissues express β3-AR mRNA at levels exceeding the detection limit in RNAseq analysis (Uhlén et al., 2015). Although β2-ARs have been localized to several regions of the human eye, β3-AR localization remains to be determined with the exception of some findings on β3-AR expression by human retinal endothelial cells and Müller cells in which β3-AR is upregulated in response to hypoxia (Steinle et al., 2003, 2005; Lucchesi et al., 2025). That β3-AR might be upregulated in clinical settings, although far from being demonstrated, is a reasonable assumption as the human β3-AR gene contains HIF-1 binding sites that allow its potential transactivation by HIF-1 (Amato et al., 2022). Previous findings support this possibility as, for instance, the fact that β3-AR levels are increased in human umbilical cord blood in concomitance with progressively decreased oxygen delivery (Scaramuzzo et al., 2026) or in human heart failure in response to sympathetic overstimulation possibly to protect the heart against hypertrophic remodeling (Michel et al., 2020).

### 4.2 β3-AR agonism prevents the neovascular response to hypoxia

As shown by the present results, β3-AR agonism would act by promoting vascular regrowth in the central avascular zone thus preventing the hypoxia-driven neovascularization in the midperiphery in line with previous findings emphasizing the need for treatments to achieve retinal reperfusion once capillaries drop out in response to ischemia (Mohite et al., 2023). There are indications that intervening in the hyperoxic phase by preventing vascular regression in the central retina or in the hypoxic phase by recovering the avascular area through HIF-1 inhibitors (Usui-Ouchi et al., 2020; Ma et al., 2025; Bailey et al., 2026) stabilizes retinal vessels and improves vascular regrowth finally sparing retinal neurons from degeneration and allowing the recovery of retinal function. Targeting the avascular area shows an apparent advantage over anti-VEGF drugs, which inhibit retinal neovascularization without affecting the vaso-obliterative phase that can even be exacerbated by anti-VEGF treatments in the OIR model (Tokunaga et al., 2014). In this respect, the recovery of hyperoxia-associated vessel loss is a major issue in ROP treatment, which is generally focused on the late stage of pathological neovascularization when major events leading to neovessel growth have already taken place (Filippi et al., 2026). On the other hand, a reduced avascular area does not necessarily mean that it has been replaced by functional vascularization unless evidence of vascular perfusion can be provided. In this respect, the finding that after β3-AR activation, Evans Blue dye remained confined within central retinal vessels, where it binds circulating serum albumin, indicates preserved vascular integrity and functional perfusion.

As shown by the present results, β3-AR agonism would act through a negative feedback mechanism, that downregulates the pro-angiogenic cascade and prevents β3-AR upregulation in response to hypoxia thus suggesting the involvement of β3-AR in a negative feedback mechanism attempting to normalize retinal vascularization. In response to BRL37344, β3-AR protein is drastically downregulated through transcriptional mechanisms as demonstrated by downregulated levels of HIF-1, which is known to transactivate the β3-AR gene (Amato et al., 2022). This is in line with the transient downregulation of β3-AR mRNA by β3-AR agonism as observed in *in vivo* and *in vitro* models (Okeke et al., 2019).

Given that hypoxia-induced β3-AR upregulation is mediated by HIF 1, inhibition of HIF 1 may be sufficient to recapitulate the effects of β3 AR agonism. In line with this hypothesis, the HIF 1α inhibitor IDF 11774 has been shown to attenuate pathological neovascularization, promote revascularization of the avascular areas, and restore BRB integrity in the OIR model through the modulation of multiple hypoxia driven angiogenic pathways, without evidence of cytotoxicity or interference with physiological angiogenesis (Yang et al., 2025). On the other hand, the clinical development of HIF-1α inhibitors has been significantly hindered by several limitations, primarily related to a lack of specificity, toxicity, and delivery challenges. In addition, besides the attractiveness of drugs targeting transcription factors, their role as convergence points of multiple signaling pathways and regulators of numerous downstream genes can lead to broad and unpredictable effects.

### 4.3 β3-AR agonism restores compromised BRB and improves ERG

As shown here, neovascular recovery by β3-AR agonism is accompanied by restored Evans Blue extravasation and improved levels of BRB markers. Of these, both ZO-1 and claudin-5 are crucial proteins, that when disrupted in OIR, affect the integrity of the BRB (Bora et al., 2023). In particular, decreased claudin-5 levels have been associated with increased vessel leakage and leakage-permissive vessels are characterized by the absence of claudin-5 (Nakamura et al., 2012) thus confirming that claudin-5 loss is a central event in determining BRB disruption. On the other hand, the OIR model is characterized by a complex regulation of tight junction proteins with some findings indicating that claudin-5 levels are not affected by OIR or are even upregulated in line with proliferating endothelial cells to form neovascular tufts (Ozawa et al., 2022; Shan et al., 2023). In this respect, agonism of β3-AR has been recently found to attenuate blood-brain barrier leakage in aged mice in which β3-AR activation has been found to mitigate brain microvascular alteration (Natarajan et al., 2024).

In the retina, restoration of BRB integrity is crucial for the maintenance of proper visual function through associated beneficial effects on both photoreceptors and post-receptor cells (O'Leary and Campbell, 2023; Zhang and Jin, 2023) in line with the fact that therapeutic interventions aimed at restoring BRB integrity may have a high impact on recovering ERG dysfunction. Vision restoration after ischemic injury is a direct consequence of the revascularization of the central retina that prevents the neovascular response to hypoxia and the concomitant BRB extravasation. In this respect, ERG assessment is the best way to evaluate at the functional level the reparative action of β3-AR activation even if treatments that counteract pathologic neovascularization may not necessarily prevent retinal dysfunction (Hatzopoulos et al., 2014).

### 4.4 β3-AR agonism prevents OIR-associated retinal damage: a potential role of astrocytes

As shown by the present results, BRL37344 recovers the damaging effects of hypoxia in association with restored astrocyte template in the central retina. In the OIR model, the hyperoxic phase causes astrocyte loss in the central retina leading to the formation of an avascular zone that creates a hypoxic environment, which in turn causes neovessel proliferation in the retinal periphery (Dorrell et al., 2010; Bucher et al., 2013; Feng et al., 2025). In the OIR model, astrocyte rescue in the avascular zone leads to normalized revascularization of the central retina (Dorrell et al., 2010). As shown by the present results, β3-AR agonism restores the astrocyte template in the central retina suggesting that β3-AR might be associated with the migration of astrocytes in the avascular zone where they facilitate recruitment and patterning of the central retinal vasculature.

As a consequence of the vaso-obliteration in the central retina, reduced oxygen levels dysregulate molecular pathways controlling astrocyte proliferation in the periphery, thereby generating excess astrocytes that contribute to pathologic vascular development (Perelli et al., 2021). Recovered astrocyte density in the central retina and restored astrocyte morphology and density in the periphery are central events to prevent pathological angiogenesis. Our finding that β3-AR activation recovers the astrocyte template in the central retina as demonstrated by GFAP-IR, is also supported by PAX2 expression that identifies both immature and mature perinatal astrocytes in which PAX2 colocalizes with GFAP both in the retina and the optic nerve (Paisleyn and Kay, 2021).

How β3-AR activation recovers the astrocyte template is difficult to hypothesize. NE released in response to hypoxia is known to activate retinal astrocytes that respond to sympathetic overstimulation through the activation of β-ARs including β3-AR (Kitano et al., 2021). β-ARs would act by promoting astrocyte process formation in line with the possibility that NE may participate in shaping astrocyte patterning, which is crucial for retinal angiogenesis (Baldwin et al., 2024). In this respect, the finding that β3-AR activation prevents OIR-associated impairment of PAX2 expression suggests the possibility that β3-AR might be involved in sculpting astrocytes into a template that guides the correct vascularization of the retina by facilitating endothelial cell migration through pro-angiogenic factors secreted by repaired astrocytes. On the other hand, the present findings from microdissected preparations suggest that β3-AR agonism might act directly on β3-AR expressed by astrocytes where it is upregulated in response to hypoxia in concomitance with GFAP upregulation. The marked reduction of β3-AR and GFAP expression following BRL37344 administration indicates that β3-AR activation may contribute to attenuating astrocyte reactivity in response to hypoxic conditions. The additional evidence that β3-AR agonism inhibits astrocyte-mediated pro-angiogenic signaling suggests a role for β3-AR in modulating the angiogenic environment to promote vascular remodeling in contrast to pathological vessel growth.

In physiological conditions, the correct formation of a vascular network is allowed by a permissive ECM environment that is in part created by astrocyte secretion of components that drive endothelial cell migration and participate in the stabilization of the vascular network (Zhang et al., 2026). Here, we provide the first evidence that β3-AR expressed by astrocytes might contribute to the formation of the retinal astrocytic template, which guides endothelial cell migration. Despite our validation of the β3-AR antibody at the astrocyte level, localization studies of β3-AR in the retina have been limited by several factors including its low expression unless in response to ischemic conditions (Ristori et al., 2011; Chen et al., 2012) and its low target selectivity as it is raised against a G protein-coupled receptor. In this respect, many β3-AR antibodies fail to display relevant target selectivity when tested with stringent criteria (Cernecka et al., 2012).

Our finding that BRL37344 almost normalizes astrocyte production of scaffolding molecules supports a role of β3-AR in regulating the interactions between astrocytes and their surrounding ECM, a prerequisite for the formation of an appropriate vascular network in the retinal superficial plexus. Among scaffolding proteins, fibronectin 1, laminins and integrins are significantly altered in OIR to dictate vascular remodeling in response to hypoxia in which their increased production by astrocytes is consistent with the formation of angiogenic processes (Puebla et al., 2022). The finding that scaffolding molecules are substantially reduced by BRL37344 suggests that β3-AR activation plays an important role in the recovery of the astrocyte template. This confirms and expands the function of β3-AR in the physiology of astrocytes in which β3-AR is known to play a role in their metabolism and protective function toward retinal neurons (Cammalleri et al., 2023; Pasha et al., 2024).

Beyond their vascular functions, astrocytes provide trophic support to retinal neurons, particularly RGCs, and the loss of trophic support is a hallmark of ischemic retinal diseases (Vecino et al., 2016; Claes et al., 2019; Zhang et al., 2026). In the GCL, astrocytes do not invest in the RGC somas but are confined to the RGC axons surrounding them in the laminar portion of the optic nerve where they release trophic factors that are essential for RGC survival. In glaucoma, a well-established model of RGC loss, remodeling of the astrocyte network may precede RGC axon damage suggesting that targeting astrocytes might help to open new perspectives in RGC protection (Lee et al., 2025). As shown by the present results, decreased β-III-tubulin immunostaining in response to hypoxia is indicative of a lower density of RGC axons in the nerve fiber layer and its recovery after β3-AR activation in concomitance with restored astrocytes suggests that β3-AR plays a critical role in the relationship between RGC axons and astrocytes.

The present findings demonstrate an association between astrocyte recovery, neurotrophic factor expression, and RGC preservation but do not establish a causal relationship. For instance, improved RGC survival/function could simply result from restored perfusion and reduced retinal ischemia rather than specifically from astrocyte-derived neurotrophic support. In addition, when considering RGC vulnerability and neuroprotection, the contribution of oxidative stress and inflammatory mechanisms to RGC dysfunction and degeneration should be taken into consideration. For instance, inflammatory responses are known to play a critical role in retinal injury and may contribute to neuronal dysfunction through the release of cytokines, chemokines, and other cytotoxic mediators by activated glial and immune cells (Amato et al., 2021). Furthermore, pharmacological interventions targeting ocular inflammation have been shown to attenuate inflammatory damage in experimental models, highlighting the potential role of inflammation in the control of retinal protection (Pittalà et al., 2015). Therefore, the beneficial effects observed in the present study may result from the combined modulation of vascular, neurotrophic, and inflammatory pathways rather than from astrocyte recovery alone. Future studies specifically addressing inflammatory and oxidative stress markers, together with selective manipulation of astrocyte-derived neurotrophic signaling, will be necessary to better define the mechanisms underlying RGC preservation.

Little is known in respect to a potential antioxidant role of β3-AR activation except for some findings demonstrating that NE increases glutathione concentration in brain astrocytes via β3-AR stimulation thus protecting them from oxidative stress-induced death (Yoshioka et al., 2021).

The present findings demonstrate that neurotrophic factors are oppositely regulated by hypoxia in both the retina and microdissected astrocytes indicating that their alterations participate in creating a hostile environment for retinal cell survival. Except for the fact that trophic factors may be produced by several retinal sources, including astrocytes, the observation that they are influenced by β3-AR agonism in both the retina and microdissected astrocytes suggests a direct role of astrocytes in mediating β3-AR-dependent retinal responses.

Of the two neurotrophic factors, CNTF levels are increased in response to hypoxia while BDNF levels are decreased. The levels of both factors almost recover their normoxic levels after β3-AR activation in an attempt to provide correct support to retinal neurons. In the case of BDNF, its reduction in response to hypoxia is consistent with impaired support to RGCs as BDNF plays a pivotal role in mediating RGC survival and preventing apoptotic signals (Abraham and Telias, 2025). In the glaucoma model, for instance, replacing BDNF levels has been shown to slow RGC degeneration (Feng et al., 2016; Osborne et al., 2018). In this line, recovered BDNF levels after β3-AR agonism may contribute to RGC resilience as demonstrated here by recovered RGC loss and the preserved amplitude of PERG as an index of RGC activity. There are no indications of β3-AR coupling to BDNF except for a model of heart failure characterized by BDNF loss in which β3-AR stimulation has been shown to replenish myocardial BDNF content to protect the heart against ischemic insult (Cannavo et al., 2023). More difficult would be to explain the finding that β3-AR agonism reduces CNTF levels, as the hypoxia-associated increase in CNTF expression has been shown to exert a neuroprotective action and to reflect a compensatory role in attenuating retinal neovascularization (Bucher et al., 2016). On the other hand, there are findings indicating that CNTF increase, despite its role in protecting photoreceptors, might cause adverse effects on retinal function (Wang et al., 2020) as also observed following long-term delivery of CNTF in patients with degenerative diseases of the retina (Birch et al., 2016). How β3-AR activation recovers the levels of neurotrophic factors remains to be established although there is some evidence of CNTF coupling to sympathetic transmission in the retina (Shi et al., 2012).

Taken together, the present findings identify β3-AR as a player in astrocytic adaptation to retinal ischemia, influencing pro-angiogenic signaling, ECM architecture, and trophic support. The concurrent regulation of these pathways by β3-AR provides further support for astrocytes as active responders to ischemia-elicited damage. Targeting astrocytic β3-AR may offer a strategy to limit aberrant angiogenesis thus preserving neurovascular integrity.

### 4.5 Study limitations

Despite the increasing success of β3-AR pharmacology in preclinical studies, the development of β3-AR ligands has been limited by several factors including their distinct recognition profile compared to that of the other two β-ARs (Vrydag and Michel, 2007; Cernecka et al., 2014). In addition, the structural differences between the human and rodent β3-AR binding pockets hinder the rational design of high-affinity ligands (Nagiri et al., 2021).

Among first-generation β3-AR agonists, both BRL37344 and CL316243 show higher potency and affinity for rodent than for human β3-ARs (Dongdem et al., 2024; Christ and Michel, 2026). In particular, BRL37344 displays some limitations including its mixed pharmacology toward β2-ARs as its effect may be blocked by β1/2-AR antagonism (Pott et al., 2003) or its coupling to α1-ARs and muscarinic receptors (Kubota et al., 2002; Matsubara et al., 2002). For its part, CL316243 shares with BRL37344 the chemical class both belonging to phenethanolamines, but it is more selective for β3-AR than β1- or β2-AR although with poor selectivity toward β2-AR (Baker, 2005). Neither BRL37344 nor CL316243 has been approved for clinical use, but BRL37344 is often used for its broader efficacy in preclinical studies (Cernecka et al., 2014).

The overview of the two agonists leads us to assume that their responses might not necessarily result from β3-AR activation warranting caution in suggesting β3-AR as a potential target for the treatment of proliferative retinopathies. As highlighted by Christ and Michel (2026), any inference on the use of β3-AR-associated therapeutic intervention must include detailed pharmacological tests such as the validation of agonist efficacy through the use of additional β3-AR agonists or β1- or β2-AR blockade. In this context, the fact that BRL37344 effects are mimicked by CL316243 supports a role for β3-AR activation in preventing the neovascular response to hypoxia. In addition, the fact that both agonists are ineffective when administered in combination with the β3-AR antagonist SR59230A might support the possibility that the retinal response to β3-AR agonism acts at β3-AR rather than through off-target effects. However, the equally limited pharmacological profile of SR59230A might complicate the interpretation of the results. In fact, SR59230A, although representing a useful tool to counteract the adverse catecholaminergic environment in the heart, has been found to display some affinity for β1-, β2 and α1-ARs (Pott et al., 2003). On the other hand, the possibility of mimicking BRL37344 or CL316243 effects using second generation β3-AR agonists might create some limitations when extrapolating data from rodent models. For instance, the myorelaxant effect of mirabegron in models of overactive bladder (OAB) has been recently questioned since either β2-AR agonism or α1-AR antagonism seems to participate in relaxation responses, although not specifically in the human bladder; therefore, it does not question its pharmacological use in OAB (Igawa et al., 2019).

Further complications regarding the use of β3-AR agonists arise from the fact that both BRL37344 and CL316243 exert systemic metabolic, cardiovascular, and urological effects due to the widespread distribution of β3-ARs in multiple tissues. Consequently, the retinal benefits observed following treatment cannot be unequivocally attributed to a direct action on retinal cells, since indirect effects mediated by changes in systemic metabolism, vascular function, or inflammatory status may also contribute to the observed phenotype. With regard to the route of administration, systemic drug administration requires high circulating concentrations to achieve therapeutic levels in the posterior segment of the eye. However, this approach may increase the risk of off-target exposure and systemic adverse effects. Consequently, local delivery strategies have been proposed as a more effective alternative. Nevertheless, drug targeting of posterior ocular tissues, including the uvea, vitreous, choroid, and retina, remains highly challenging due to the presence of specialized anatomical and physiological barriers that protect the eye from pathogens, foreign bodies, and toxic agents. Following topical administration, drug delivery to the posterior segment of the eye remains highly inefficient, with only a small fraction of the applied dose, estimated at approximately 5%, reaching the target tissues via either the corneal or the conjunctival-scleral route. Despite these limitations, considerable efforts are currently focused on the development of advanced drug delivery systems designed to enhance the penetration and bioavailability of topically administered therapeutics in the posterior eye. Although these emerging strategies have shown promising results, the majority are still in the preclinical stage of development (Ye et al., 2025). An additional complication is the use of the 129S strain. The rationale for using this strain is quite reasonable, as previous studies have demonstrated marked upregulation of β3-AR expression under pathological conditions, making it a suitable model for investigating the contribution of β3-AR signaling. Nonetheless, this characteristic may also represent a limitation. The increased receptor expression characteristic of this mouse strain may increase sensitivity to β3-AR agonists, potentially amplifying treatment effects but limiting the translational value of the present findings. Consequently, the efficacy of β3-AR activation might be different from that observed in other mouse strains, which differ in adrenergic receptor expression, metabolic regulation, inflammatory responses, and retinal disease susceptibility. This issue is particularly relevant from a translational perspective, since human retinal tissues may not exhibit the same degree of β3-AR upregulation as the 129S strain. Therefore, the potential therapeutic efficacy of β3-AR activation that can be extrapolated from the 129S strain could be overestimated compared to what could be achieved in other groups with more heterogeneous genetic backgrounds. Further experiments will aim to replicate the present results in additional mouse strains. In fact, models with greater translational relevance will be necessary to establish the translational value of potential β3-AR-associated therapeutic strategies.

## 5 Conclusion

The finding that β3-AR agonism acts on the vaso-obliterated phase of OIR is particularly attractive as the severity of vaso-obliteration largely influences subsequent neovascular processes suggesting that β3-AR may be a novel and promising therapeutic target for ROP treatment. On the other hand, interventions that promote revascularization of the central retina are not expected to proportionally affect the neovascular process while reducing neovessel proliferation does not necessarily promote physiologic vessel regrowth into the avascular area. On the other hand, the attribution of the observed effects specifically to β3-AR activation requires greater caution particularly in the absence of experimental designs pointing to underline a causality between β3-AR activation and RGC restoration. The additional finding that the β3-AR-associated recovery of the avascular area occurs in concomitance with improved astrocyte template and that astrocyte responses to hypoxia are directly affected by β3-AR activation suggests that β3-AR agonism protects the hypoxic retina by restoring the repair mechanisms managed by astrocytes. Although the use of β3-AR agonists might be facilitated by the fact that they are already present on the market for treating patients suffering from OAB in which they are well tolerated at the eye level (Cicek et al., 2025) and the fact that a non-randomized trial is already ongoing to determine if β3-AR agonists, used in patients with OAB and concurrent age-related macular degeneration (AMD), can safely and effectively reduce the conversion of eyes from dry to wet AMD (Ambrosio et al., 2026), further studies are needed to establish a causal relationship between β3-AR agonism and retinal rescue from hypoxic damage.

## Data Availability

The raw data supporting the conclusions of this article will be made available by the authors, without undue reservation.
