# Supplementary material for: Novel insights into β3-adrenoceptor function in a model of retinopathy of prematurity: the contribution of astrocytes to retinal protection
Source: Front Neurosci. 2026 Aug 19;20:1891523. doi: 10.3389/fnins.2026.1891523 (PMC13533968; doi:10.3389/fnins.2026.1891523)
Supplement: Supplementary file 1 [file Data_Sheet_1.pdf]

Supplementary Figures

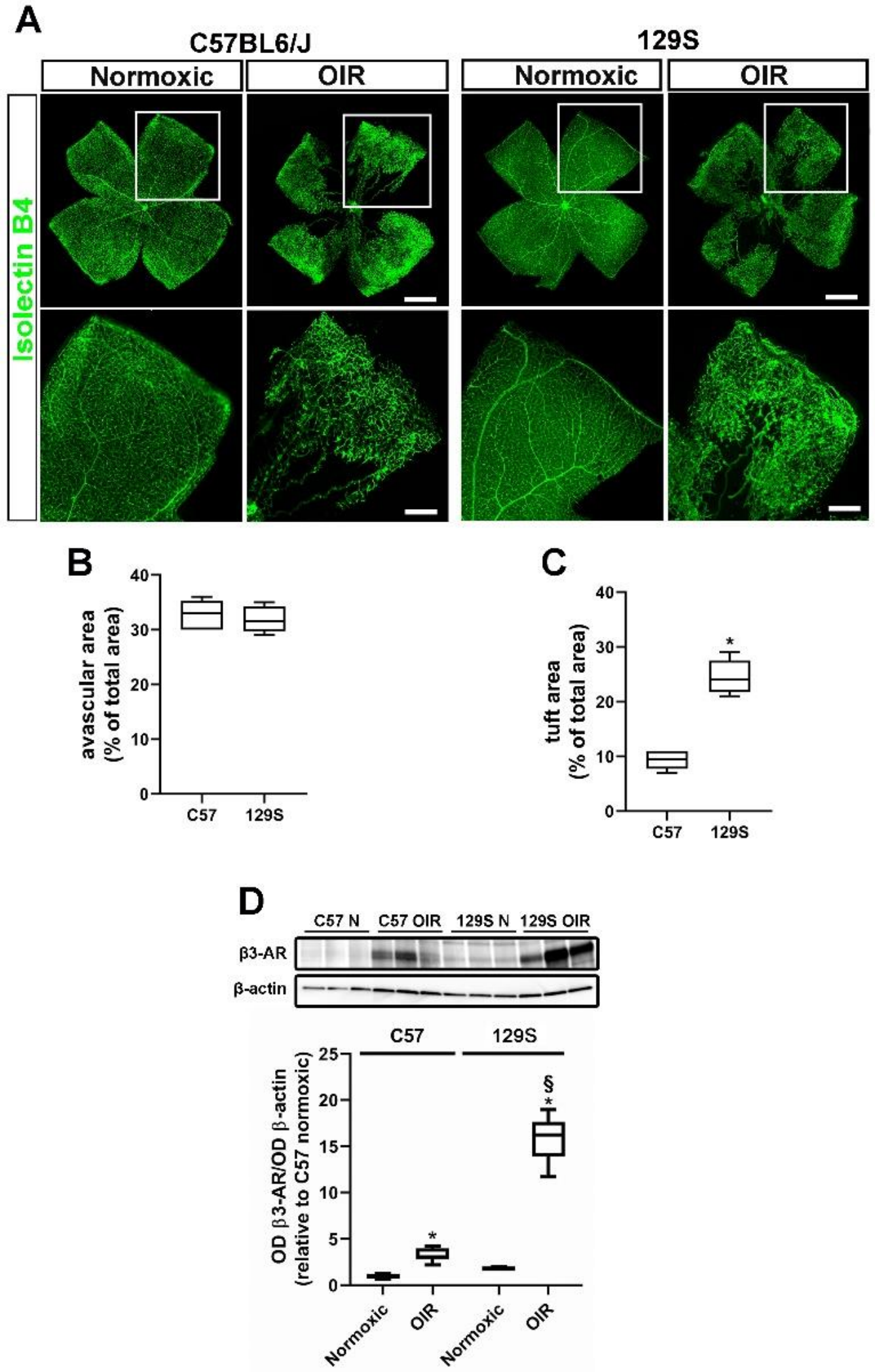

Supplementary Figure 1. Higher responsiveness to hypoxia by the 129S strain. (**A**) Representative images of retinal whole mounts labeled with isolectin B4 (IB4) from normoxic and oxygen-induced retinopathy (OIR) mice of the C57 and the 129S strains including high magnification of the boxed areas (scale bar: 1 mm in the whole mounts and 500 µm in the boxed areas). (B-C) The extent of the avascular area (**B**) and the tuft area (**C**) was quantitatively evaluated in OIR mice of both strains. Data are shown as box plots with minimum to maximum whiskers (n = 6 retinas). In **D**, representative Western blots and relative densitometric analysis of β3-adrenoceptor (-AR) protein levels in retinal homogenates from postnatal day (PD) 17 mice of the C57 strain versus the 129S strain. β-actin was used as a loading control. Data are shown as box plots with minimum to maximum whiskers (n = 6 samples). *P < 0.05 vs normoxic C57; §P< 0.05 vs normoxic 129S.

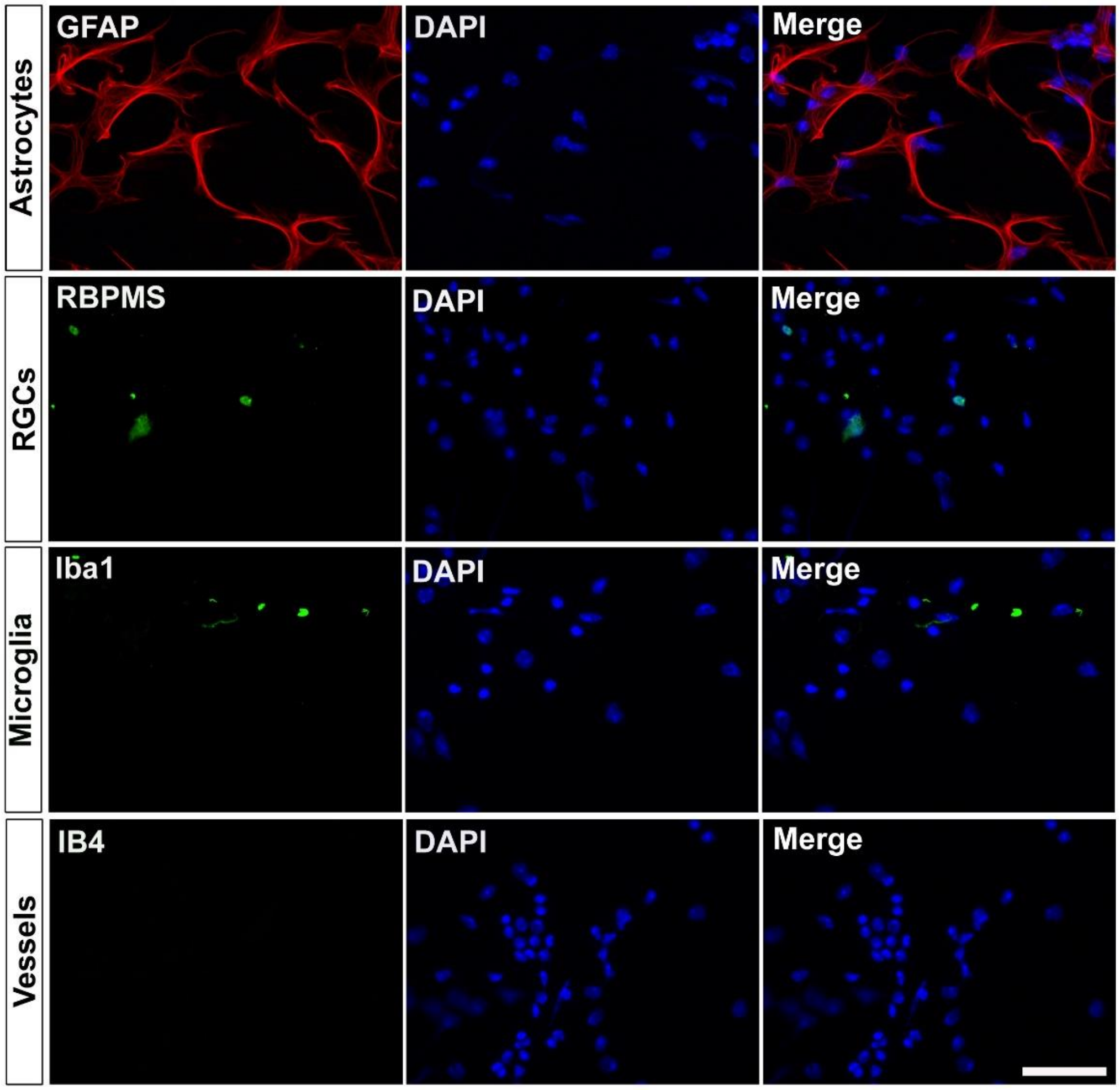

Supplementary Figure 2. Validation of the pull-off technique leading to microdissected astrocytes. Representative images of microdissected astrocytes stained with different markers including antibodies against GFAP to evidentiate astrocytes, RBPMS to label RGCs and Iba1 to label microglial cells. IB4 staining was used to evidentiate retinal vessels. DAPI was used for nuclear staining in fluorescence microscopy. Scale bar: 50 µm.

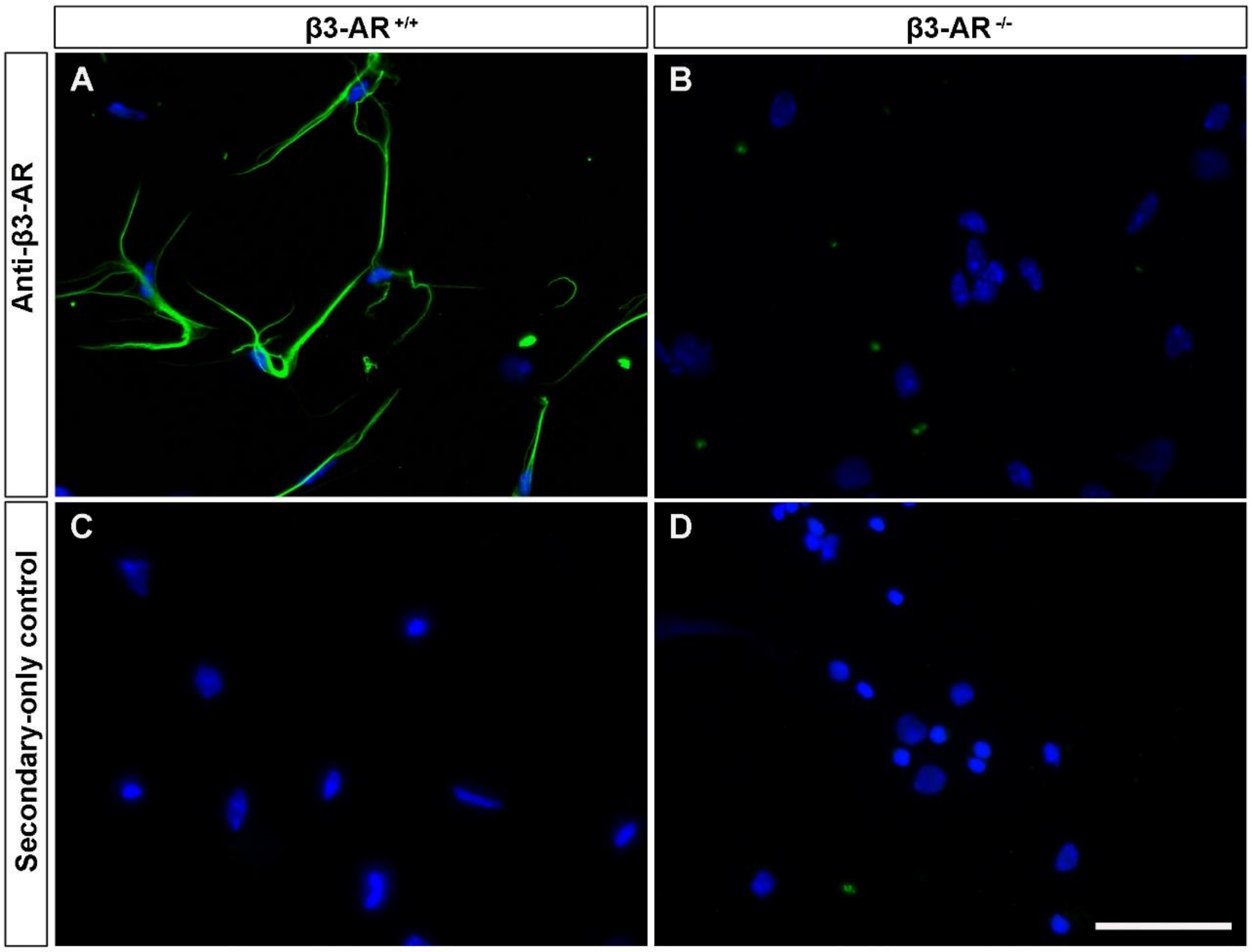

Supplementary Figure 3. Validation of the β3-AR antibody. Representative images of microdissected astrocytes immunostained with antibodies against β3-AR in β3-AR$^{+/+}$ and β3-AR$^{-/-}$ mice (**A,B**). **C,D** secondary-only controls. DAPI was used for nuclear staining in fluorescence microscopy. Scale bar: 50 µm.
